# Identification of combinatorial miRNA panels derived from extracellular vesicles as biomarkers for esophageal squamous cell carcinoma

**DOI:** 10.1002/mco2.377

**Published:** 2023-09-18

**Authors:** Yaojie Wang, Xiaoya Li, Xiaojian Wei, Lei Li, Hanyu Bai, Xi Yan, Hongtao Zhang, Libo Zhao, Wei Zhou, Lianmei Zhao

**Affiliations:** ^1^ Research Center The Fourth Hospital of Hebei Medical University Shijiazhuang China; ^2^ Key Laboratory of Tumor Gene Diagnosis, Prevention and Therapy of Hebei Province Shijiazhuang China; ^3^ Hangzhou Institute of Medicine (HIM) Chinese Academy of Sciences Hangzhou China; ^4^ University of Pennsylvania School of Medicine Philadelphia Philadelphia Pennsylvania USA

**Keywords:** esophageal squamous cell carcinoma, extracellular vesicle, liquid biopsy, lymph node metastasis, miRNAs

## Abstract

MicroRNAs (miRNAs) are relatively stable in blood, emerging as one of the most promising biomarkers in tumor liquid biopsy. Both total and extracellular vesicles (EVs) encapsulated miRNA have been studied for prognostic potential in a variety of cancers. Here, we systematically compared and verified the total and vesicle‐derived miRNA expression profiles from plasma samples in healthy controls and patients with esophageal squamous cell carcinoma (ESCC). In the present study, four miRNA species miR‐636, miR‐7641, miR‐28‐3p, and miR‐1246 that were differentially expressed in ESCC patients were chosen for further study. We first elucidated their essential function in ESCC progression and further explored their preliminary mechanism by identifying target proteins and involving signal pathways. Subsequently, the prognostic miRNA panels including miR‐636, miR‐7641, miR‐1246, and miR‐28‐3p for ESCC diagnosis were constructed and validated using different cohort. Our results showed that the panel including the above four miRNAs derived from plasma EVs was most effective in distinguishing tumor patients from normal subjects, while integrated plasma EVs‐derived miR‐1246, miR‐28‐3p and total plasma miRNAs miR‐636, miR‐7641 showed the best capability in predicting lymph node metastasis. In summary, our studies revealed that plasma EVs‐derived miRNAs could be emerged as promising biomarkers for ESCC diagnosis.

## Graphical abstract

We firstly chosed four miRNAs that differentially expressed in ESCC patients, then elucidated their essential function in ESCC progression and explored their target genes. Furthermore, the prognostic miRNA panel including miR‐636, miR‐7641, miR‐1246, and miR‐28 for ESCC diagnosis, and the predictive model including EVs‐derived miR‐1246, miR‐28‐3p and total plasma miR‐636, miR‐7641 for lymph node metastasis were constructed

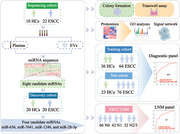

## 1 INTRODUCTION

Esophageal cancer ranks seventh in global cancer incidence and sixth among the causes of cancer‐related death worldwide, with a 5‐year survival rate ranging from 20 to 30%.^1^, ^2^ More than half of the new cases and deaths occur in China,^3^ and over 90% of patients suffer from esophageal squamous cell carcinoma (ESCC).^1^, ^4^ Gastroscopy and biomarker testing are currently the most popular diagnostic methods for ESCC, while in low sensitivity and specificity for early‐stage ESCC patients. It is urgent to develop new ESCC biomarkers and detection methods for early and advanced diagnosis to improve the survival rate of ESCC patients.

In recent years, liquid biopsy including the analysis of circulating tumor cells, circulating tumor nucleic acid/protein, and the extracellular vesicles (EVs) in the body fluids, has become a promising technique for tumor diagnosis. Of which, EVs is one of the nanoscale particles packed in the membrane, containing a series of biomolecules secreted by cells, such as protein, lipids, and nucleic acid. Additionally, EVs are relatively stable in various biological liquids and tissues, playing an important role in intercellular communication by transferring functional RNAs and proteins.^5^, ^6^, ^7^, ^8^ Therefore, EVs have been widely considered novel diagnostic biomarkers for the progression of malignant tumors. For example, the research found that the cell surface proteoglycan, glypican‐1 (GPC‐1) was specifically enriched on EVs derived from cancer‐cell,^9^ and the GPC‐1 alone or in combination with growth factor receptor or epithelial cell adhesion molecules, could be used to diagnose pancreatic ductal adenocarcinoma.^10^ In another study, the expression level of circ‐IARS was significantly upregulated in plasma‐derived EVs of patients with metastatic pancreatic cancer, and positively correlated with liver metastasis and TNM stage.^11^

Meanwhile, microRNA (miRNA), which is a quantitative dominant RNA cargo of EVs, has emerged as a feasible diagnostic and prognostic biomarker in ESCC. For example, a study confirmed that the plasma miR‐21 expression levels were significantly higher, while the plasma miR‐31 and miR‐375 expression levels were significantly lower in ESCC patients compared with healthy controls (HCs), thus the plasma‐derived miR‐21, miR‐31, and miR‐375 could be potential biomarkers for the diagnosis of ESCC.^12^ In the part of vesicle‐free miRNA which is mainly associated with the argonaute protein family, EV‐derived miRNAs have also been recognized as promising biomarkers for disease diagnosis. For example, plasma EV‐derived miR‐125a‐3p was significantly increased in colorectal cancer, which has an important diagnostic ability for early colorectal cancer.^13^ In addition, it has been shown that elevated levels of serum EV‐derived miRNA‐182, and miRNA‐766‐3p in ESCC patients were positively associated with poor prognosis.^14^, ^15^ A similar study found that downregulation of serum EV‐derived miRNA‐652‐5p was associated with lymph node metastasis (LNM).^16^ However, it is unclear whether miRNAs from different source have similar or significantly different performances in the diagnosis of ESCC.

In the present study, we performed a next‐generation sequencing (NGS) for the aberrant miRNAs in plasma or plasma‐derived EVs from the first cohort including Healthy controls (HCs, *n* = 10) and ESCC patients (*n* = 22). Through statistical analysis of the expression level of candidate miRNAs in plasma and plasma‐derived EVs, we established an ESCC diagnostic panel (plasma EV‐derived miRNAs: miR‐636, miR‐7641, miR‐1246, and miR‐28‐3p) and a LNM diagnostic panel including total plasma miRNAs (miR‐636 and miR‐7641) and plasma EV‐derived miRNAs (miR‐1246 and miR‐28‐3p) based on miRNAs expression level and area under the receiver operating characteristic (ROC) curve (AUC) value, then we verified the above two panels using two independent cohorts. Additionally, this study investigated whether the same miRNA in different forms (total plasma miRNAs or plasma EV‐derived miRNAs) represented diverse biological meanings and diagnostic efficiency for ESCC, raising that it is worthy to consider including both forms of miRNA markers in a single detection model to achieve optimal diagnosis performance.

## 2 RESULTS

### 2.1 Isolation and verification of plasma‐derived EVs

**FIGURE 1:**
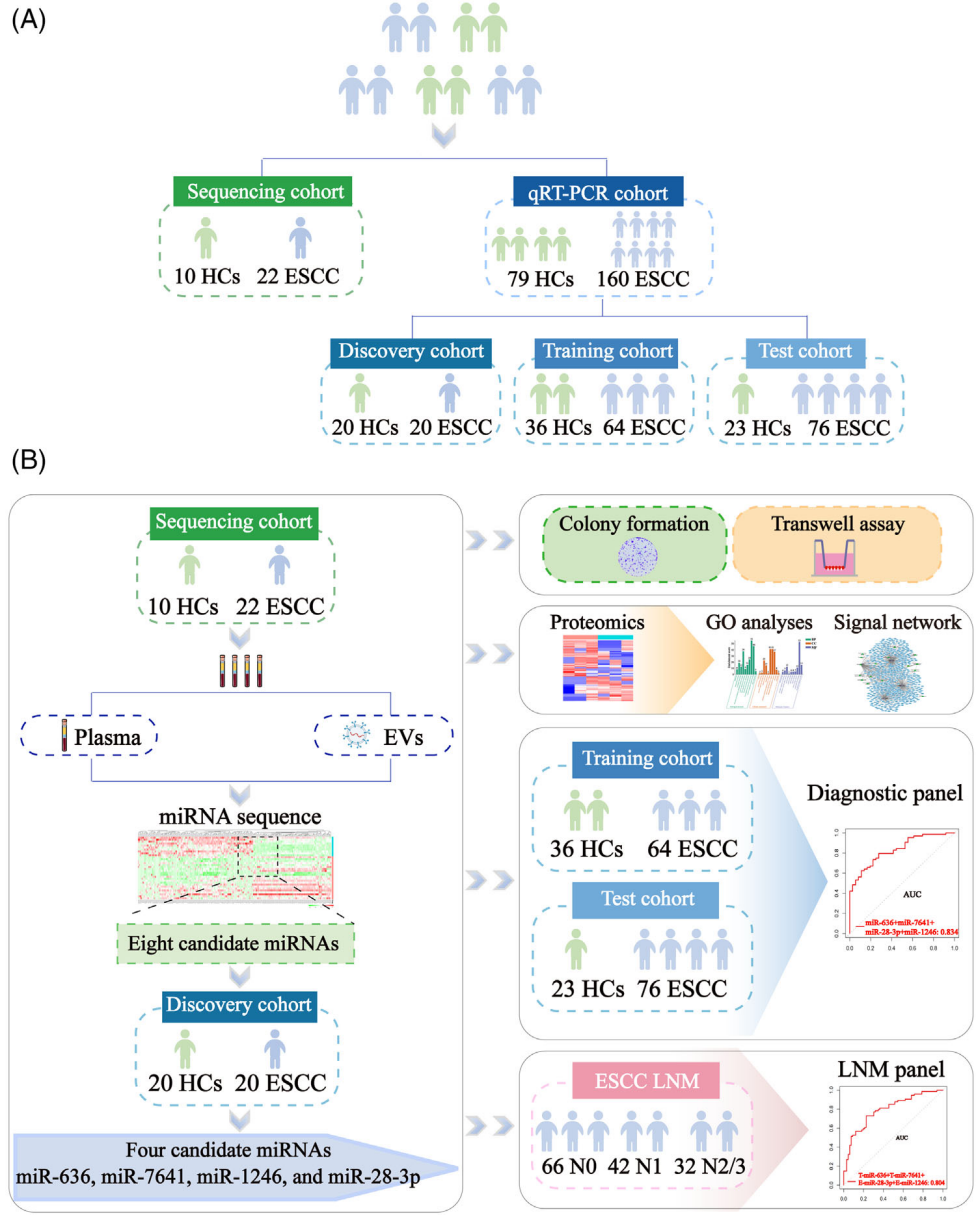
Schematic overview of the experimental workflow. (A) The design of the miRNA sequencing cohort and qRT‐PCR cohort; the qRT‐PCR cohort consists of discovery cohort, training cohort, and test cohort. (B) The flow diagrams show the screening of candidate miRNAs, the biological functions of miRNAs, the finding of target genes and prediction of binding sites, and the construction of diagnostic models. The flow diagrams were drawn by Adobe Illustrator software.

**FIGURE 2:**
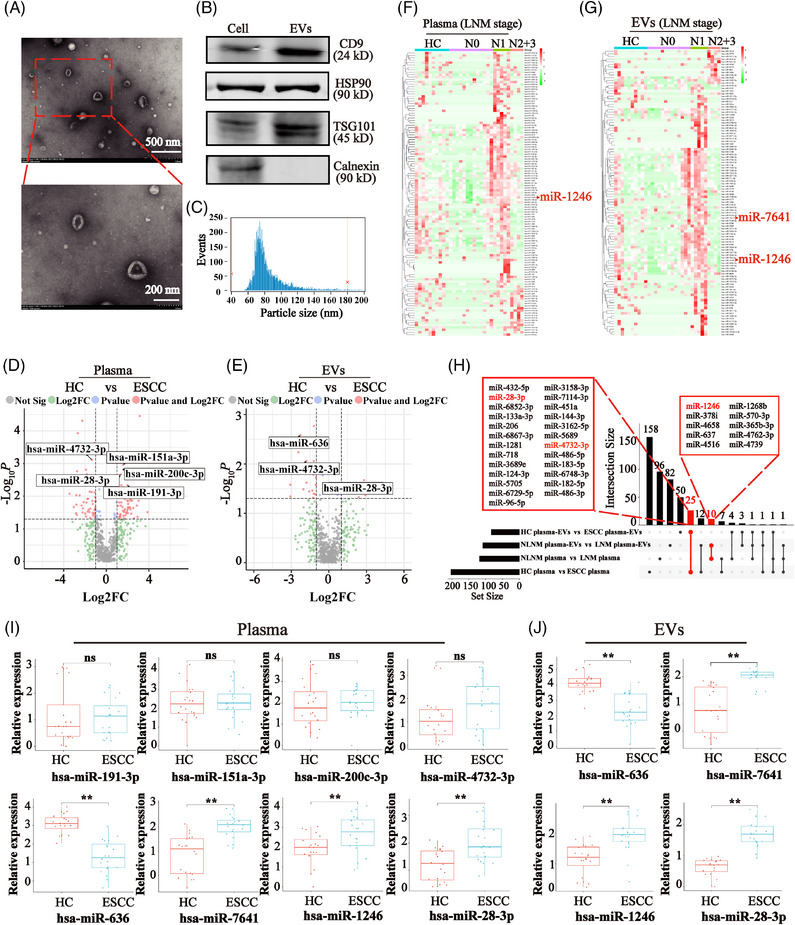
Screening of candidate miRNAs for ESCC diagnosis. (A) Images of transmission electron microscopy showing that EVs are bowl‐shaped. Scale, 200 and 500 nm. (B) Western blot analysis showing the expression of CD9, HSP90, and TSG101, and the absence of Calnexin. (C) Nano‐FCM analysis indicating the size distribution of EVs; mean size was 71.63 nm. (D and E) The volcanic map of abnormal miRNAs (HC *vs*. ESCC) in plasma (B) and plasma‐derived EVs (C). Red dots indicate higher‐ or lower‐abundance miRNAs in ESCC patients. FC > 1.5 or FC < 0.67, and *p* < 0.05. (F and G) Heatmap of differentially expressed miRNAs (HC *vs*. N0 *vs*. N1 *vs*. N2/3) in plasma (D) and plasma‐derived EVs (E). FC > 1.5 or FC < 0.67, and *p* < 0.05. (H) Four comparisons were shown in the UpSet plot. The numbers on the bars show the numbers of overlapping miRNAs in the corresponding comparison. (I) The expression levels of selected total plasma miRNAs in the discovery cohort (miR‐191‐3p, miR‐151a‐3p, miR‐200c‐3p, miR‐4732‐3p, miR‐636, miR‐7641, miR‐1246, and miR‐28‐3p). (J) The expression levels of candidate plasma EV‐derived miRNAs in the discovery cohort (miR‐636, miR‐7641, miR‐1246, and miR‐28‐3p).

To probe the miRNA in plasma and plasma‐derived EVs from ESCC patients, we establish a tested queue that includes a miRNA sequencing cohort (22 ESCC patients and 10 HCs) and a reverse‐transcription and quantitative real‐time PCR (qRT‐PCR) cohort (160 ESCC patients and 79 HCs); of these, the qRT‐PCR cohort consists of three cohorts: discovery cohort (20 ESCC patients and 20 HCs), training cohort (64 ESCC patients and 36 HCs), and test cohort (76 ESCC patients and 23 HCs), as detailed shown in Figure 1A and Table S1. Then the plasma and plasma‐derived EVs were subjected to miRNA sequence between HC and ESCC, and ESCC with or without LNM, as shown in Figure 1B. To investigate the expression profile of miRNAs in plasma‐derived EVs in ESCC, we firstly extracted the EVs from the plasma of ESCC patients and HCs by combining size‐exclusion chromatography (SEC) and ultracentrifugation (UC) method,^17^ and characterized these samples by transmission electron microscopy (TEM), nano‐flow cytometry (Nano‐FCM), and western blotting according to the MISEV2018 guidelines.^18^ Accordingly, the typical cup‐shaped morphologies were observed in plasma‐separated particles (Figure 2A). Furthermore, the EVs‐specific protein markers CD9, HSP90, and TSG101 were all detected in the particles isolated from plasma, while Calnexin, an endoplasmic reticulum protein being a negative EVs marker,^19^ was not observed (Figure 2B). In addition, the size of plasma‐separated particles ranged from 40 to 176 nm with an average of 71.63 nm (Figure 2C), consistent with the size of EVs in guidelines.^18^ The above results proved that EVs were successfully separated from plasma.

It was reported that the EVs contain a different spectrum of RNAs than plasma.^20^ Therefore, the distribution of different types of RNA including miRNA, ribosomal RNA, and transfer RNA was compared in plasma and plasma‐derived EVs from 22 ESCC and 10 HCs. As revealed by the NGS result, different RNA distribution was shown between EVs and plasma, and the miRNA content in plasma was significantly higher than that in plasma EVs (Figure S1A). Furthermore, to explore the existence form of miRNA in plasma and plasma‐derived EVs, we analyzed 20 samples for the proportion of EV‐derived miRNAs in total miRNAs. The results showed that about 3.3–12.7% of miRNAs were vesicle associated (Figure S1B).

### 2.2 Identification of candidate miRNAs as biomarkers for ESCC

We then focused on differentially expressed miRNA profiles in plasma and plasma‐derived EVs. Our analysis revealed that 82 upregulated miRNAs and 122 downregulated miRNAs in ESCC plasma as compared with those in HCs (Figures 2D and S2A, and Table S2). In addition, 14 miRNAs were upregulated, whereas 69 were downregulated in ESCC plasma‐derived EVs as compared with those in HCs (Figures 2E and S2B, and Table S3).

In addition, we also established the miRNAs expression profiles from ESCC patients with different LNM stages, which included 118 abnormally expressed miRNAs in plasma (Figure 2F and Table S4) and 108 differential miRNAs in plasma‐derived EVs (Figure 2G and Table S5). Through comprehensive analysis, this differential miRNA profile included 25 miRNAs which were identical to those in plasma or plasma‐derived EVs of ESCC, and 10 miRNAs coexisting in plasma and plasma‐derived EVs of ESCC with LNM (Figure 2H, Tables S6 and S7). Among these miRNAs, three miRNAs were overexpressed in ESCC plasma (miR‐151a‐3p, miR‐200c‐3p, and miR‐191‐3p), and miR‐636 was decreased in ESCC plasma‐derived EVs. Additionally, miR‐28‐3p and miR‐4732‐3p were overexpressed and decreased, respectively, in both ESCC plasma and plasma‐derived EVs. Furthermore, miR‐1246 was overexpressed in both plasma and plasma‐derived EV of ESCC with LNM. In addition, as miR‐7641 was significantly increased in the plasma‐derived EVs of ESCC with LNM (Table S5), and had been shown to promote tumorigenesis and chemoresistance in several malignant tumors,^21^, ^22^ thus it was also selected as a candidate biomarker for subsequent study.

To validate the robustness of miRNA sequencing, the levels of eight candidate miRNAs were initially verified by Quantitative Real‐time PCR (qRT‐PCR) in the discovery cohort, which included 20 HCs and 20 ESCC patients. The qRT‐PCR results confirmed that miR‐636 was significantly decreased in the total plasma of ESCC, while miR‐7641, miR‐1246, and miR‐28‐3p were significantly increased in the plasma of ESCC patients compared with HCs. However, the miR‐191‐3p, miR‐151a‐3p, miR‐200c‐3p, and miR‐4732‐3p had no significant difference (Figure 2I). Consistently, plasma EV‐derived miR‐636 was significantly decreased, whereas the miR‐7641, miR‐1246, and miR‐28‐3p were significantly upregulated in plasma‐derived EVs of ESCC patients (Figure 2J). Based on the above results, we selected miR‐636, miR‐7641, miR‐1246, and miR‐28‐3p as the candidate biomarkers for further verification.

Furthermore, to determine whether the EV‐derived miRNAs we detected indeed came from the plasma‐derived EVs, we examined the expression levels of miR‐7641 in the 10 fractions isolated by the SEC method. The result showed that the miR‐7641 was mainly enriched in EVs associated fractions 2, 3, and 4, suggesting that miR‐7641 was mainly from plasma‐derived EVs (Figure S3A). In addition, as miRNAs from plasma‐derived EVs are resistant to RNases and Proteinases, we treated the EV samples isolated from the plasma with RNase A and Proteinase K. After treatment, the levels of the four plasma EV‐derived miRNAs were not significantly changed as revealed by the qRT‐PCR results (Figure S3B). Taken together, these candidate miRNAs in EVs that we detected indeed mainly exist in the form of EVs encapsulated.

### 2.3 The biological role of candidate miRNAs

In another, Pearson's correlation analysis was performed to examine the correlation of four candidate miRNAs derived from plasma and plasma EVs, and the result showed that miR‐636 derived from EVs showed a significant negative correlation with miR‐7641, consistent with the qRT‐PCR validation results; however, there was no significant correlation between the level from plasma EVs‐derived miRNAs and plasma‐derived miRNAs, indicating that there might be significant differences in the expression and diagnostic efficacy of plasma miRNAs versus plasma EVs‐derived miRNAs for disease (Figure S3C).

**FIGURE 3:**
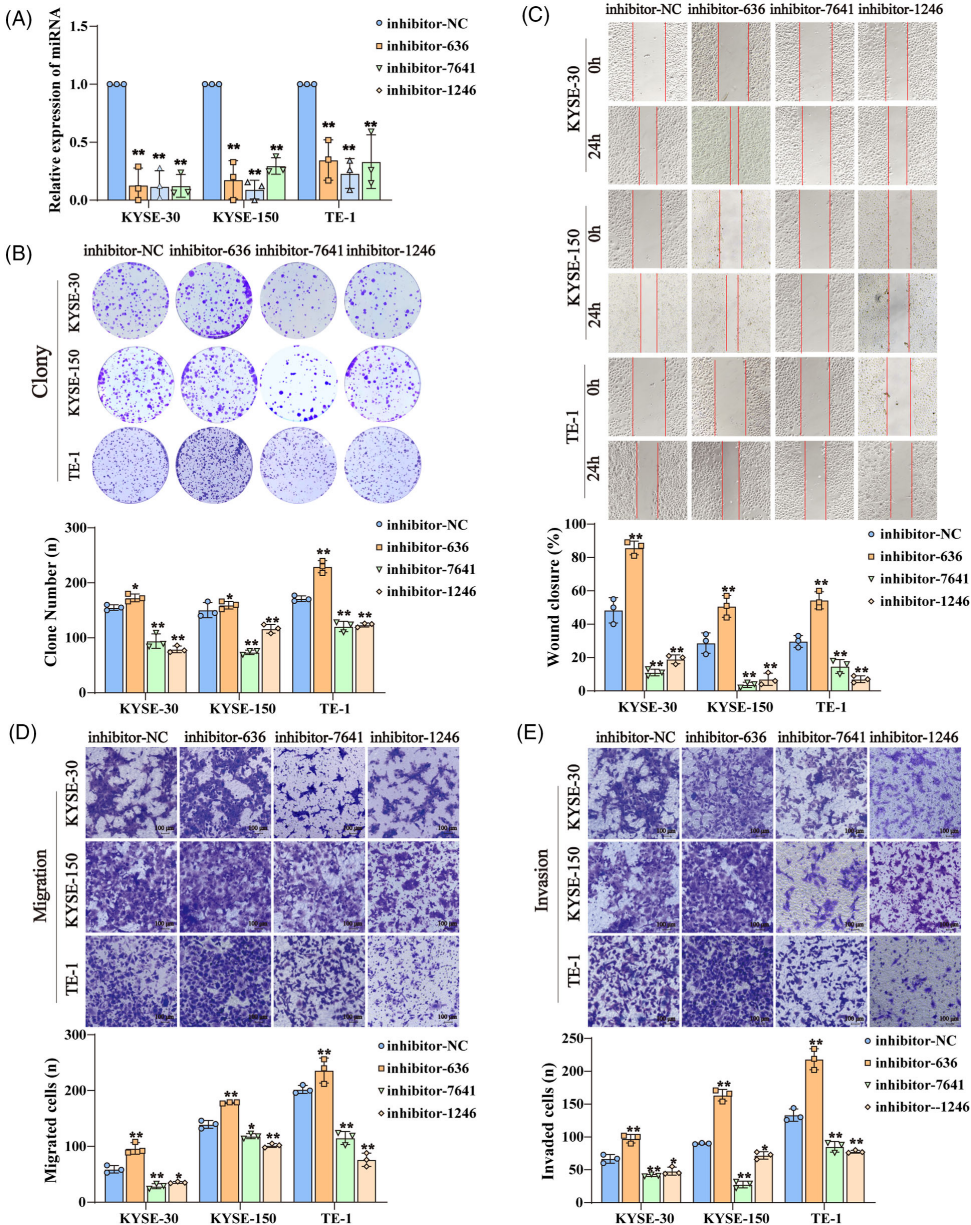
The biological role of candidate miRNAs. (A) The expression of miR‐636, miR‐7641, and miR‐1246 were measured by qRT‐PCR in KYSE‐30, KYSE‐150, and TE‐1 cells transfected with the miRNA inhibitors for 24 h. (B) The colony‐formation ability of ESCC cells knocking down miRNA was assessed by clonogenic assay. The number of colonies was calculated (*n* = 3) and plotted on a histogram. (C) The wound‐healing assay showed the effect of miRNA knockdown on cell mobility of ESCC cells. (D and E) The effect of miRNA knockdown on the migration (D) and invasion (E) of ESCC cells was investigated using the Transwell and Matrigel assays, respectively. Average counts were collected from three random microscopic fields. *p < 0.05 and **p < 0.01 versus the control groups.

To investigate the role of these candidate miRNAs in ESCC development, we examined the effect of these miRNAs on the migration, invasion, and clonogenicity of ESCC cells by modulating miRNA levels. Out of these four candidates, miR‐28‐3p had been reported to promote tumor cell proliferation and metastasis in a variety of tumors including ESCC.^23^, ^24^, ^25^, ^26^ Thus, we focused on investigate the function of miR‐636, miR‐7641, and miR‐1246 in ESCC cells. The results showed that knocking down miR‐636 by siRNA (Figure 3A) could promote the ability of colony formation (Figure 3B), migration (Figures 3C and D), and invasion (Figure 3E); however, knocking down miR‐7641 or miR‐1246 significantly reduced the ability of colony formation, migration, and invasion in ESCC cells. On the contrary, overexpression of miR‐636 significantly decreased the ability of colony formation, migration, and invasion, and overexpression of miR‐7641 or miR‐1246 could increase the ability of colony formation, migration, and invasion in ESCC cells (Figure S4). Altogether, the above results demonstrate that miR‐636, miR‐7641, and miR‐1246 could be involved in regulating the clonogenicity, migration, and invasion of ESCC cells.

### 2.4 Analysis of target genes and involved signaling pathway of candidate miRNAs

**FIGURE 4:**
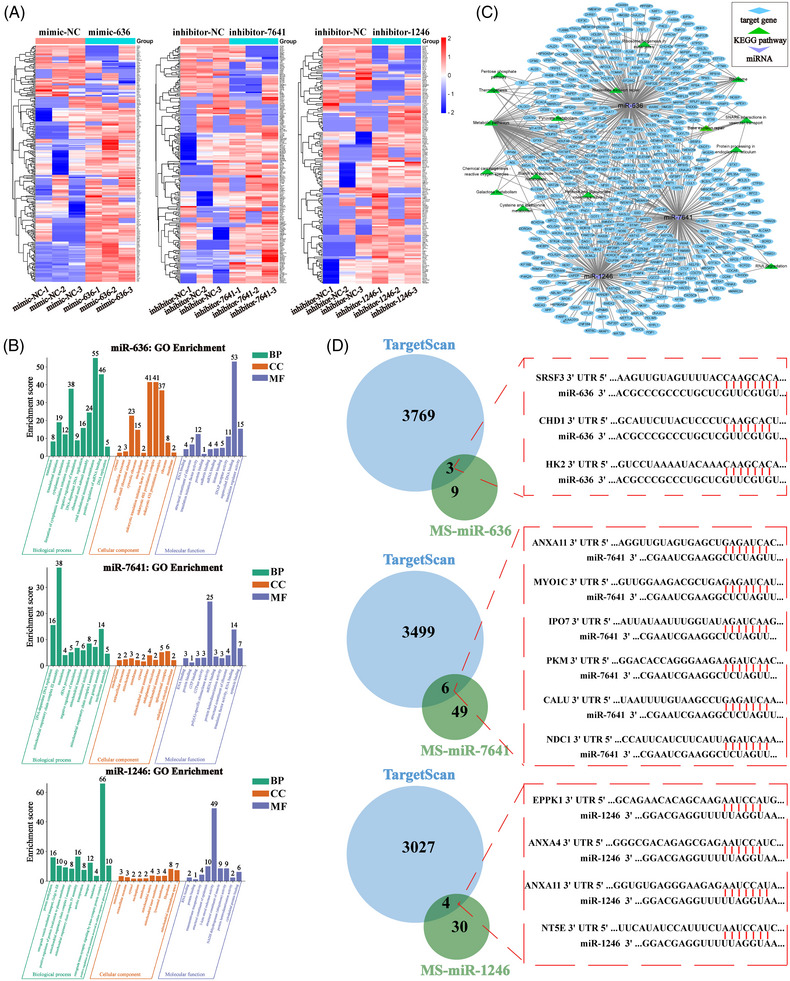
Analysis of target genes and involving signaling pathway of candidate miRNAs. (A) Heatmap of all differentially expressed proteins by miR‐636, miR‐7641, and miR‐1246 detected by proteomics. FC > 1.2 or FC < 0.8 and *p* < 0.05. (B) GO analysis on the biological processes (BP), cellular components (CC), and molecular functions (MF) of differentially expressed proteins of miR‐636, miR‐7641, and miR‐1246. (C) The signaling network diagram for the differentially expressed proteins of miR‐636, miR‐7641, and miR‐1246. (D) A Venn diagram was used to investigate the common target gene for each group (TargetScan, our data). In total, in the intersection of the Venn diagram, three target genes were identified by miR‐636, six target genes were identified by miR‐7641, and four target genes were identified by miR‐1246.

We further evaluate the underlying mechanism of miRNAs modulating the ESCC. First, we performed proteomics to identify the target proteins in miR‐636‐overexpressing, miR‐7641‐knocking down, and miR‐1246‐knocking down ESCC cells using the threshold of fold change (FC) > 1.2 or FC < 0.8, *p* < 0.05 (Figure 4A). The Gene Ontology (GO) analysis and Kyoto Encyclopedia of Genes and Genomes (KEGG) enrichment analysis of these differentially expressed proteins were performed using DAVID (The Database for Annotation, Visualization, and Integrated Discovery).^27^ The most enriched categories and the enrichment scores in biological process, cellular component, and molecular function were shown in Figure 4B. As revealed by the results, we observed that differentially expressed proteins affected by overexpression of miR‐636 were mainly enriched in the category of positive regulation of mRNA binding, formation of cytoplasmic translation initiation complex, and ribosomal small subunit assembly; furthermore, the differentially expressed proteins in the cells after miR‐7641‐knocking down were mainly enriched in the category of DNA‐dependent DNA replication, mitochondrial respiratory chain complex III assembly, and stress granule assembly; besides, the differentially expressed proteins in the cells with downregulation of miR‐1246 were mainly enriched in the category of retrograde trans‐synaptic signaling by a trans‐synaptic protein complex, mitochondrial respiratory chain complex IV assembly, and protein methylation. Additionally, we further tested the signaling network with the Cytoscape software. As revealed by the result, the enrichment pathways targeted by these three candidate miRNAs were mainly involved in the metabolic, chemical carcinogenesis‐reactive oxygen species, and ribosome pathway (Figure 4C).

Furthermore, it is known that miRNAs can exert regulatory effects on target mRNAs by destabilizing them or inhibiting their translation.^28^ Therefore, we selected the differentially expressed proteins that downregulated in miR‐636 (FC < 0.7, *p* < 0.05), and those upregulated proteins (FC > 1.2, *p* < 0.05) in miR‐7641 and miR‐1246 as the potential target genes for further analysis. Additionally, we predicted the target genes of three miRNA through the online website: TargetScan (https://www.targetscan.org/vert_80/)^29^ and compared them with our data obtained from proteomics. As a result, 3, 6, and 4 target genes of miR‐636, miR‐7641, and miR‐1246 were identified after Venn diagram analysis of the two groups, and the binding sites were shown in Figure 4D and Table S8. The above research preliminary explores the biological functions and signaling pathways of proteins targeted by miR‐636, miR‐7641, and miR‐1246, providing the basis for subsequent studies of miRNA mechanisms.

### 2.5 Construction of miRNA biomarker panels for ESCC

**FIGURE 5:**
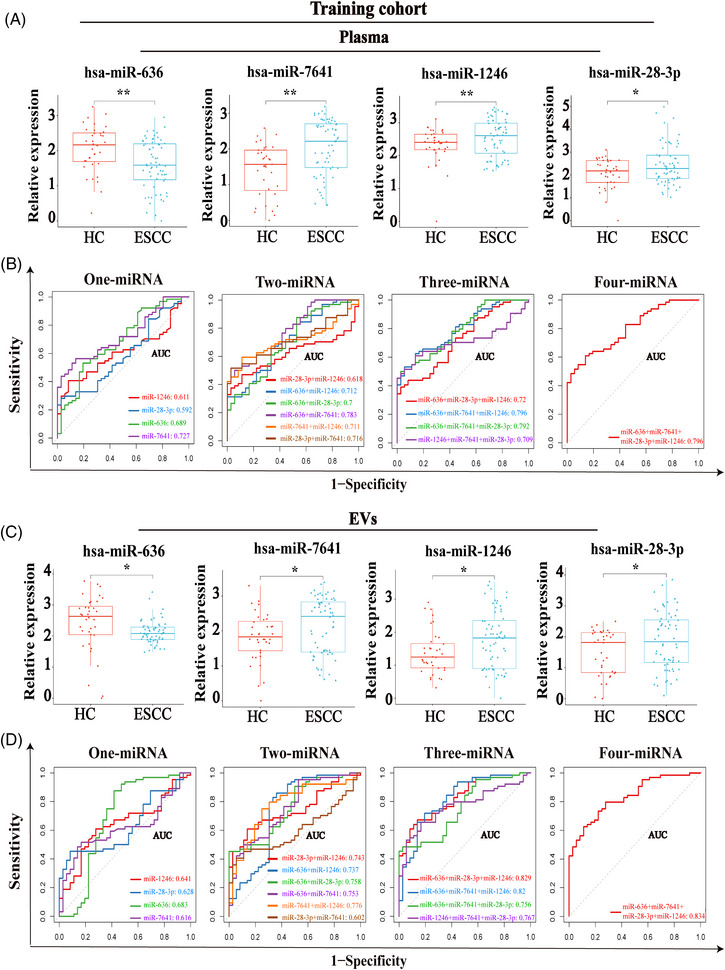
Construction of miRNAs‐based panels for ESCC diagnosis. (A) Expression levels of candidate total plasma miRNAs (miR‐636, miR‐7641, miR‐1246, and miR‐28‐3p) in the training cohort. (B) ROC curves of the 1−4 total plasma miRNAs‐based model in the training cohort. (C) Expression levels of selected plasma EV‐derived miRNAs (miR‐636, miR‐7641, miR‐1246, and miR‐28‐3p) in the training cohort. (D) ROC curves of the 1−4 plasma EV‐derived miRNAs‐based model in the training cohort.

Next, to construct miRNAs‐based panels for ESCC diagnosis, we examined levels of four candidate miRNAs in a training cohort including 100 plasma samples (64 ESCC *vs*. 36 HCs). Consistent with the screening results, the level of total plasma miR‐636 was significantly downregulated, whereas levels of total plasma miR‐7641, miR‐1246, and miR‐28‐3p in ESCC patients were significantly upregulated as compared with that in the plasma from HCs (Figure 5A). Furthermore, the logistic regression analysis revealed that a total plasma miRNA panel comprising miR‐636, miR‐7641, miR‐28‐3p, and miR‐1246 had the best performance in the ROC analysis (AUC: 0.796) (Figure 5B).

We also assessed the diagnostic efficacy of miRNAs in plasma‐derived EVs by analyzing levels of candidate miRNAs with the same training cohort. Consistently, as compared with HCs, the level of miR‐636 was decreased, and levels of miR‐7641, miR‐1246, and miR‐28‐3p were all increased in the plasma‐derived EVs of ESCC patients (Figure 5C). Here we identify plasma EVs‐derived miR‐636, miR‐7641, miR‐1246, and miR‐28‐3p that exhibited an AUC of 0.683, 0.616, 0.641, and 0.628, respectively, to distinguish ESCC from HCs. Many studies considered circulating miRNA could serve as a biomarker of ESCC and most of them reported an AUC around 0.7,^30^ similar to our results. In addition, numerous studies have demonstrated that the combination of multiple miRNAs can achieve higher diagnostic efficacy than single miRNAs in tumor patients.^31^, ^32^, ^33^, ^34^ Therefore, we combined four plasma EVs‐derived miRNA for the diagnosis of ESCC, and the combination model showed optimum diagnostic capabilities for the detection of ESCC (AUC = 0.834), which was higher than that of total plasma miRNA panels (Figure 5D and Table S9). Thus, combining plasma EV‐derived miR‐636, miR‐7641, miR‐1246, and miR‐28‐3p model had better diagnostic efficiency for ESCC.

### 2.6 Validation of miRNAs combination panels for ESCC diagnosis

**FIGURE 6:**
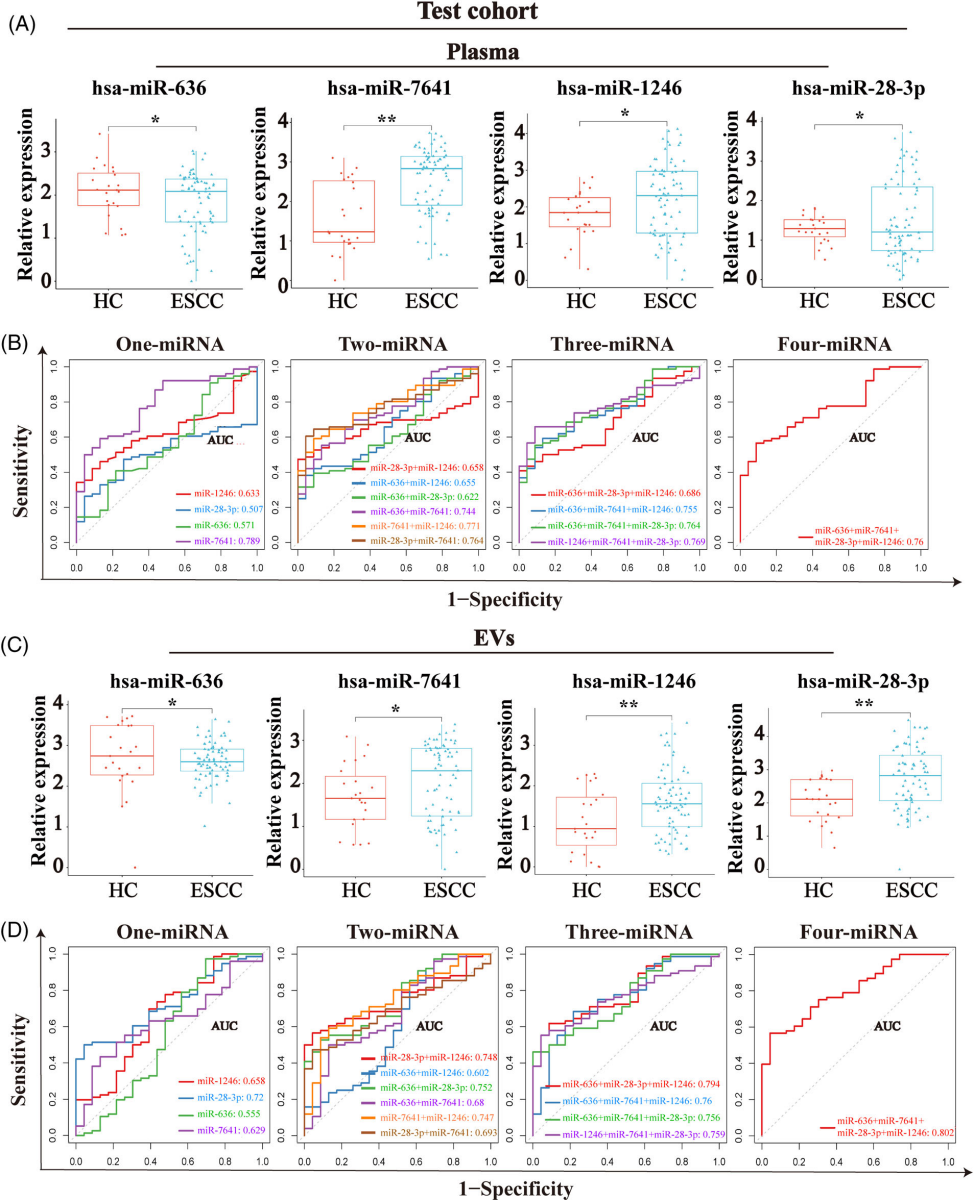
Validation of miRNAs‐based panels for ESCC diagnosis. (A) Expression levels of selected total plasma miRNAs (miR‐636, miR‐7641, miR‐1246, and miR‐28‐3p) in the test cohort. (B) ROC curves of the 1−4 total plasma miRNAs‐based model in the test cohort. (C) Expression levels of selected plasma EV‐derived miRNAs (miR‐636, miR‐7641, miR‐1246, and miR‐28‐3p) in the test cohort. (D) ROC curves of the 1−4 plasma EV‐derived miRNAs‐based model in the test cohort.

To further verify the four candidate miRNAs and miRNAs combination panels for ESCC diagnosis, we performed a diagnostic performance analysis in another independent test cohort containing 76 ESCC patients and 23 HCs. Consistent with the results in the training cohort, the level of miR‐636 was significantly reduced, while the levels of miR‐7641, miR‐1246, and miR‐28‐3p were significantly increased both in plasma (Figure 6A) and plasma‐derived EVs of ESCC patients (Figure 6C). However, the highest AUC was exhibited in the panel containing only miR‐7641 in plasma (AUC: 0.789) (Figure 6B). In plasma‐derived EVs, the highest AUC was still the panel consisting of four candidate miRNAs (AUC: 0.802) (Figure 6D and Table S9). Thus, the performance of miRNAs panel in plasma‐derived EVs is higher for ESCC diagnosis than that in plasma.

### 2.7 Identification of candidate miRNAs in different stages of LNM

**FIGURE 7:**
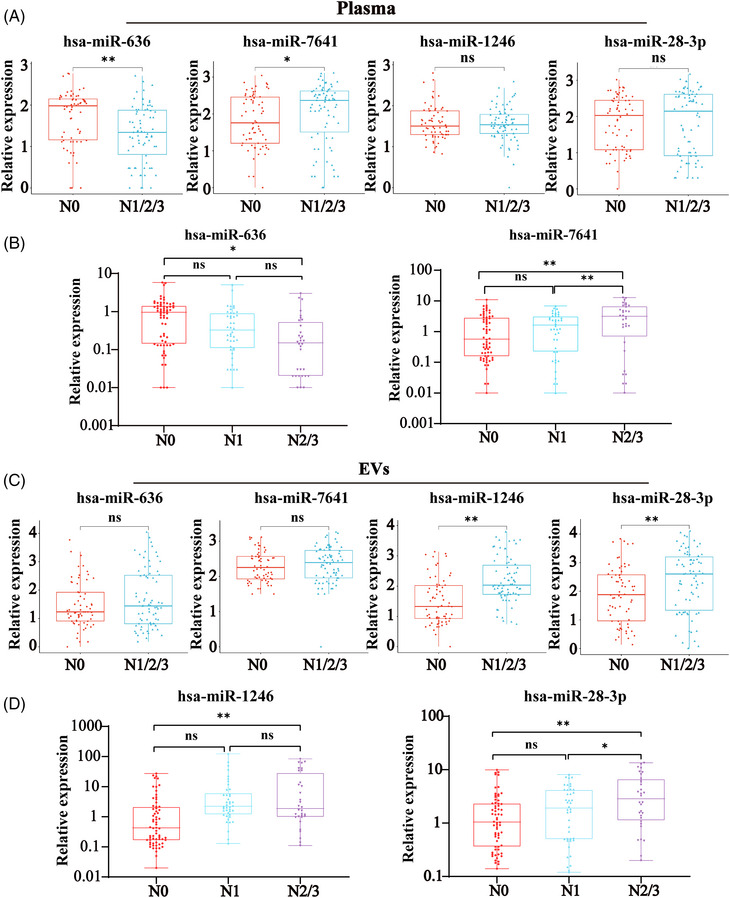
Validation of candidate miRNAs in different stages of LNM. (A) Expression of total plasma miR‐636, miR‐7641, miR‐1246, and miR‐28‐3p in ESCC patients with LNM (N1/N2/N3) and without LNM (N0). (B) Expression levels of total plasma miR‐636 and miR‐7641 in ESCC patients with different LNM stages (N0 *vs*. N1 *vs*. N2/N3). (C) Expression levels of plasma EV‐derived miR‐636, miR‐7641, miR‐1246, and miR‐28‐3p in ESCC patients with LNM (N1/N2/N3) and without LNM (N0). (D) Expression levels of plasma EV‐derived miR‐1246, and miR‐28‐3p in ESCC patients with different LNM stages (N0 *vs*. N1 *vs*. N2/N3). *p < 0.05 and **p < 0.01 versus the control groups.

Diagnosis of LNM is essential for the planning of treatments for ESCC patients. Thus to identify a miRNA biomarker panel for LNM, we performed an additional analysis based on 140 ESCC patients’ clinical outcomes, including 66 patients at the N0 stage, 42 patients at the N1 stage, and 32 patients at N2 or N3 stage, to search for candidate miRNAs. The results showed that the level of miR‐636 was significantly downregulated in the plasma of LNM patients, while the level of miR‐7641 was upregulated as a contrast (Figure 7A). In addition, the level of total plasma miR‐7641 could significantly distinguish the different stages of N0, N1, and N2/3 in ESCC patients, while miR‐636 only has the lower expression in N2 and N3 stage samples compared with that in N0 stage samples (Figure 7B).

Furthermore, in plasma‐derived EVs, the levels of miR‐1246 and miR‐28‐3p were significantly upregulated in LNM patients as compared with nonmetastatic patients (Figure 7C). Additionally, the plasma EV‐derived miR‐28‐3p could significantly distinguish the different stages of N0, N1, and N2/3 in ESCC patients (Figure 7D). These results suggested that total plasma miR‐636, miR‐7641, and plasma EV‐derived miR‐1246, miR‐28‐3p might be as new candidate biomarkers in diagnosing the LNM of ESCC.

### 2.8 Construction of miRNAs‐based panel for diagnosis of LNM in ESCC

**FIGURE 8:**
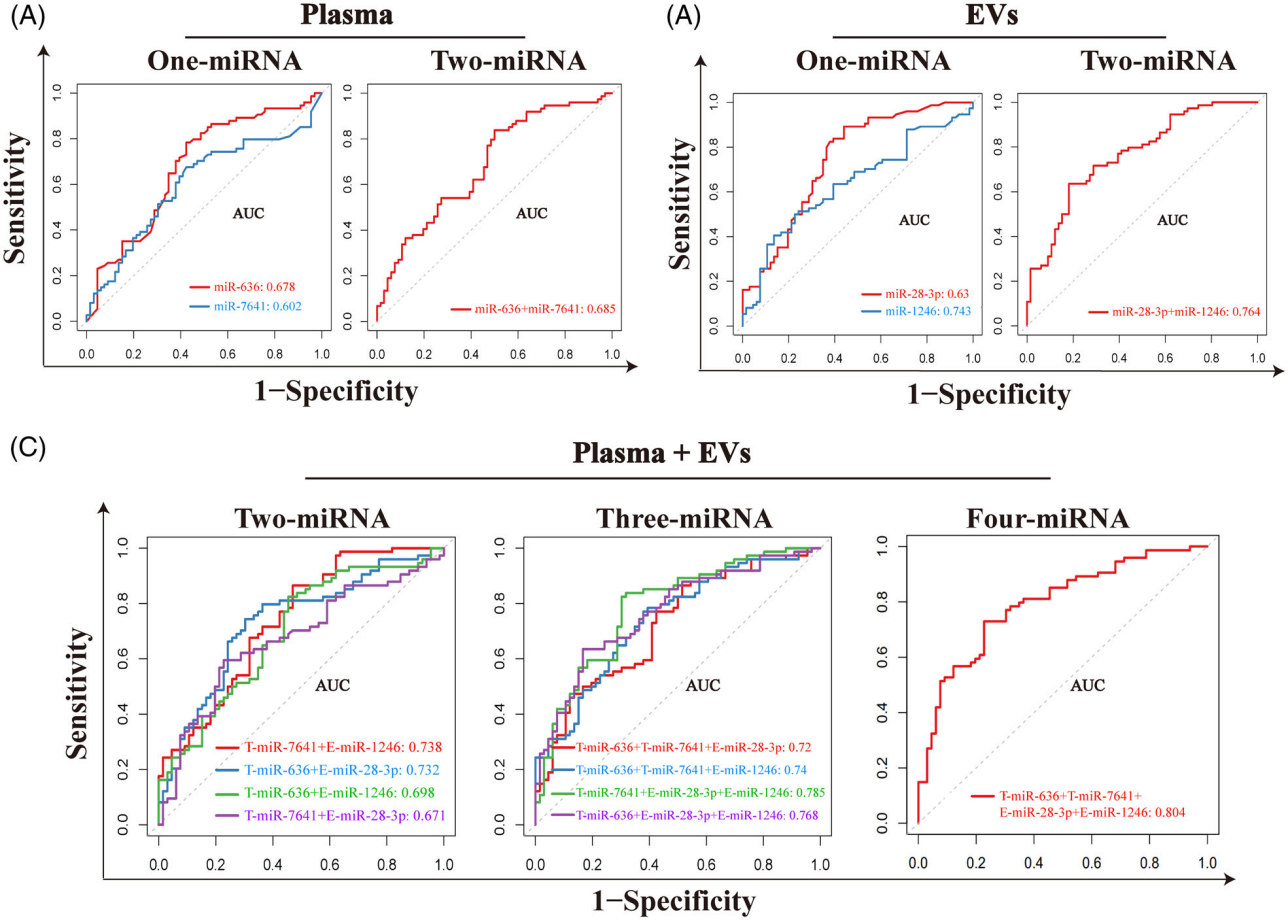
Construction of candidate miRNAs‐based panel for diagnosis of LNM in ESCC. (A) ROC curves of total plasma miRNA (miR‐636 and miR‐7641)‐based panels for the diagnosis of LNM. (B) ROC curves of plasma EV‐derived miRNA (miR‐1246 and miR‐28‐3p)‐based panels for the diagnosis of LNM. (C) ROC curves of models integrated total plasma miRNAs (T‐miR‐636 and T‐miR‐7641) with their plasma EV‐derived miRNAs (E‐miR‐1246 and E‐miR‐28‐3p) for the diagnosis of LNM. T, total; E, EVs.

Subsequently, based on the above qRT‐PCR results, we next constructed diagnostic panels for ESCC with LNM using the expression levels of total plasma miR‐636, miR‐7641, and the plasma EV‐derived miR‐1246, miR‐28‐3p. As shown in Figure 8A, the total plasma miR‐636 and miR‐7641 exhibited an AUC of 0.678 and 0.602, respectively, and the panel containing total plasma miR‐636 and miR‐7641 achieved an AUC of 0.685. Thus, the panel consisting of two plasma miRNAs is more efficient in the diagnosis of LNM then the single one. In addition, for plasma‐derived EVs, the AUC of plasma EV‐derived miR‐28‐3p and miR‐1246 exhibited 0.63 and 0.743, respectively, and the AUC of the combination of the two miRNAs achieved 0.764 (Figure 8B), supporting above result that the panel containing two plasma EV‐derived miRNAs has the better diagnostic efficacy than single plasma EV‐derived miRNAs and any total plasma miRNAs‐based panels.

To further improve the diagnostic performance of miRNA panels for LNM, we performed a logistic analysis with total plasma miRNAs (T‐miR‐636 and T‐miR‐7641) and plasma EV‐derived miRNAs (E‐miR‐1246 and E‐miR‐28‐3p). The results showed that the combination of total plasma miR‐7641 and plasma EV‐derived miR‐1246 exhibited an AUC of 0.738 (Figure 8C, left), and the AUC of three candidate miRNAs (T‐miR‐7641, E‐miR‐1246, and E‐miR‐28‐3p) was higher than the two‐miRNAs panel (Figure 8C, middle, AUC: 0.785), while the panel comprising all candidate miRNAs had the highest AUC of 0.804 (Figure 8C, right). Taken together, the above results indicated that integrating the total plasma miRNA‐based models with plasma EV‐derived miRNA‐based models could improve the diagnostic efficiency of ESCC patients with LNM (Table S10). It also implies that attempts to combine different forms of biomarkers may enhance the diagnostic efficacy of the disease to varying degrees.

## 3 DISCUSSION

Liquid biopsy is an important breakthrough in the field of tumor diagnosis and treatment.^35^ Tumors‐derived circulatory nucleic acid and protein showed obvious advantages in the early diagnosis, testing, and treatment of tumors.^36^ Among them, miRNAs play an important role in the malignant process and drug resistance of tumors. For example, circulating miR‐202‐3p was significantly upregulated in type I gastric neuroendocrine tumors and it could serve as a potential diagnostic biomarker for type 1 gastric neuroendocrine tumors.^37^ In addition, miRNAs play an essential role in ESCC diagnosis. For instance, Zeng et al. demonstrated that serum EVs‐derived miRNA‐19b‐3p levels were elevated in ESCC patients compared with healthy individuals, implying the significance of serum EVs‐loaded miRNA‐19b‐3p in the early diagnosis of ESCC.^38^ In this study, we found that overexpressing miR‐636, knocking down miR‐7641 or miR‐1246 inhibited the malignant progression of ESCC. Furthermore, the level of total plasma miR‐636 was significantly decreased, and the levels of miR‐7641, miR‐1246, and miR‐28‐3p were significantly increased in ESCC patients. In addition, the total plasma miR‐636 was significantly downregulated, while total plasma miR‐7641 was significantly upregulated in ESCC patients with LNM compared with those without LNM samples. The levels of miR‐636 and miR‐7641 in plasma may be biomarkers for the diagnosis of ESCC patients with LNM.

EVs were first found in sheep reticulocytes, which can protect miRNAs from the influence of RNase; moreover, it has the advantages of low immunogenicity and cytotoxicity, high stability, and biocompatibility.^39^ Recently, EVs had attracted much attention as a new minimally invasive biomarker in the early diagnosis and treatment of tumors.^40^ The EVs separated from plasma consist of numerous diverse classes of nucleic acids; of these, miRNA has been extensively studied in biomarkers because of its abundance and stability.^41^ For example, the combination of EV‐derived miR‐320 and miR‐574‐3p could distinguish patients with glioblastoma from healthy individuals, and the combined application could be used as a biomarker for the diagnosis of glioblastoma^42^; moreover, Min et al.^41^ found integrating four EV‐derived miRNAs together can better distinguish patients with early colorectal cancer from controls with various gastrointestinal symptoms. Furthermore, this study also revealed a significant difference between the total plasma miRNAs and plasma EV‐derived miRNAs in predicting colon cancer.^41^ In our study, we found the level of miR‐636 was decreased, and the levels of miR‐7641, miR‐1246, and miR‐28‐3p were increased in the plasma‐derived EVs of ESCC patients, and the miRNAs panel in plasma‐derived EVs is more efficient for ESCC diagnosis than that in plasma. It may be related to the mechanism of miRNAs sorting and packaging into EVs. Accumulating evidence indicates that EVs can load miRNAs selectively by a specific process.^43^, ^44^, ^45^ In a study, Liao et al.^46^ found significant differences of miRNAs in human esophageal cancer cells and EVs, further demonstrating the ability of different miRNAs to sort into EVs actively was significant difference. In short, total plasma miRNAs and plasma EV‐derived miRNAs are two completely different kinds of tumor biomarkers.

In our study, the AUC was used to evaluate the diagnostic efficacy of different standard (AUC: 0.9–1.0 was optimal; AUC: 0.8–0.9 was good; AUC: 0.7–0.8 was poor; and AUC: 0.5 deemed the result invalid). Our findings demonstrated that the four plasma EV‐derived miRNA‐based model showed optimum diagnostic capabilities for the detection of ESCC, with an AUC of 0.834, higher than the traditional tumor biomarkers we know, such as SCC‐Ag (0.665),^47^ CEA (0.549), and so on,^48^ and other miRNA‐based diagnostic markers for ESCC, for example, miRNA‐718 (0.715),^49^ miR‐216b (0.756),^50^ and so on. Additionally, the combined model of plasma total miRNA and plasma EV‐derived miRNA showed better diagnostic efficacy for LNM than the single miRNA panel, with an AUC of 0.804, higher than the conventional diagnostic techniques for lymph node metastases in other reports, such as PET/CT (0.745), MRI (0.697), CECT (0.58), and so on.^51^ Based on the above, combined plasma EV‐derived miR‐636, miR‐7641, miR‐1246, and miR‐28‐3p had better diagnostic efficiency for ESCC, and the panel integrating total plasma miRNA with plasma EV‐derived miRNA could improve the diagnostic performance for LNM in different degrees. Although the extent of improvement is minimal, this work suggested that the physiological or pathological information that may be reflected by different forms of miRNAs in the diagnosis of the same disease may not be entirely consistent. Therefore, combining two categories of miRNA biomarkers can probably provide novel insight for further development and enhancement of diagnostic tools.

However, our study still has some limitations. For example, additional studies are still required to identify whether the combination of more miRNAs can improve the diagnostic efficacy. Moreover, whether the LNM metastasis prediction panel we constructed is also applicable in the diagnosis of other tumors is unclear, and further validation is still needed. Second, the number of samples was relatively small and there was lack of long‐term outcome data, thus the larger prospective clinical trials are needed to validate these results. Furthermore, we need to establish the standardized methods for EV extraction from plasma to validate these findings and integrate total miRNA and vesicle‐derived miRNA to promote diagnostic applications in ESCC patients.

## 4 MATERIALS AND METHODS

### 4.1 Blood collection and plasma acquisition

Blood samples were collected from the Fourth Hospital of Hebei Medical University. The demographic and clinicopathological features of the 182 patients with ESCC and 89 healthy volunteers were obtained from the clinical and pathological records. The HCs were excluded if they had any history of diabetes mellitus, severe immune alterations, esophageal disease, lung disease, renal or hepatic dysfunction, malignancy, and cardiovascular event in the past 6 months.

This research was granted approval by the ethics committee of the Fourth Hospital of Hebei Medical University (approval No. 2022KS034). Before collecting the blood samples, an informed written statement was achieved from all patients with ESCC and HCs.

### 4.2 Isolation of plasma‐derived EVs

The 1 mL plasma samples were filtered via a 0.22 μM filter (SLGPR33RB; Millipore). The supernatant was loaded into a Sepharose‐based CL‐2B column (Echo9101A‐5 mL; Echobiotech). The EVs particles were separated by PBS (P1020; Solarbio). Five hundred microliters of outflow was determined as a fraction. The 2−4 fractions were collected. Next, the effluents were ultracentrifuged at 150,000×*g* for 4 h. The pellet was resuspended by PBS and ultracentrifuged again at 150,000×*g* for 2 h. In the end, the EVs was resuspended with PBS.

### 4.3 Characterization of plasma‐derived EVs

EVs isolated from plasma via combining SEC with UC were characterized under the instruction of MISEV2018 by TEM, nano‐FCM, and western blotting.^18^

#### 4.3.1 Transmission electron microscopy

Ten microliters EVs was added to Formvar‐carbon electron microscopy grids. The grids were cleaned using sterile distilled water and excess water was removed with the filter papers. Subsequently, the EVs enriched fraction was contrasted with the uranyl acetate solution. After that, we removed the excess fluid in the grids and dried them for 2 min. Finally, the morphology of EVs was recorded by the transmission electron microscope (Hitachi HT7800; Hitachi Limited).

#### 4.3.2 Nano‐FCM analysis

The detailed operating procedures of Nano‐FCM (U30; Nanofcm) were as described in the producer's instruction. Nano‐FCM was parameter calibration with the standard sample before use. The particle diameter and concentration of EV samples were analyzed based on the standard samples.

#### 4.3.3 Protein extraction and Western blot analysis

The RIPA buffer containing PMSF (R0010; Solarbio) was added to an EV sample. The protein concentration was measured using a BCA Protein Assay Kit (No. 23225; Thermo Scientific). After detecting the protein concentration, the loading buffer was added to each EVs protein sample, and boiled in a metal bath at 100°C for 10 min. The 12% sodium dodecyl sulfate‐polyacrylamide gel (SDS‐PAGE) was used to separate the total protein (60 μg) and transferred to polyvinylidene fluoride membranes (ISEQ00010; Millipore). Subsequently, the 5% nonfat milk powder in Tris Buffered Saline with 0.1% Tween‐20 (TBST) was performed to block the membranes. The membranes were subsequently incubated with the appropriate primary antibodies against CD9 (1: 200, sc‐13118; Santa Cruz), TSG101 (1: 200, sc‐7964; Santa Cruz), HSP90 (1: 2000, 13171‐1‐AP; Proteintech), and Calnexin (1: 200, sc‐23954; Santa Cruz) overnight. Following washing three times with TBST, the membranes were incubated with fluorochrome‐labeled secondary anti‐rabbit/anti‐mouse IgG for 1 h. The membranes were finally examined under the Odyssey Infrared Imaging System (9120; LI‐COR).

### 4.4 RNase A and Proteinase K treatment of EVs enriched fractions

Combined Protease K (RT403; TIANGEN) and RNase A (ST578; Beyotime) to treat the RNA derived from EVs. After treating EVs separated from plasma with 100 μg/mL of proteinase K at 37°C for 30 min, the EVs samples were incubated at 90°C for 10 min to inactivate proteinase K. The samples were then treated with 10 μg/mL of RNase A at 37°C for 15 min. The level of miRNAs was detected using qRT‐PCR.

### 4.5 RNA extraction and sequencing

RNA was extracted from plasma and plasma‐derived EVs respectively using the miRNeasy® Mini kit (217004; Qiagen). Sequencing libraries were built via a QIAseq miRNA Library Kit (331505; Qiagen). Next, the quality of the library was detected with Agilent Bioanalyzer 2100 and qRT‐PCR. Following cluster generation, the library preparations were sequenced on an Illumina HiSeq platform.

### 4.6 Reverse‐transcription and quantitative real‐time PCR

The miRNAs were reverse transcribed into cDNA via the Primer‐Script™ RT reagent kit (RR037A; TAKARA). The target gene was amplified by qRT‐PCR using the TaqMan™ probe kit (RR390A; TAKARA). RNA expression was normalized to U6. The primers purchased from Sangon Biotech Co, Ltd. and shown in Table S11.

### 4.7 Cell lines and cell culture

The ESCC cell line TE‐1 was purchased from the Shanghai Institute of Cell Biology (Chinese Academy of Sciences, Shanghai, China). KYSE‐30 cell line was kindly provided by Professor Masatoshi Tagawa (Department of Molecular Biology and Cancer Biology, Chiba University, Japan). KYSE‐150 cell line was donated by Qimin Zhan's laboratory from the Cancer Hospital of the Chinese Academy of Medical Sciences (Beijing, China). All cells were cultured in Roswell Park Memorial Institute (RPMI)‐1640 medium (C11875500BT, GIBCO, USA) supplemented with 10% heat‐inactivated fetal bovine serum (FBS) (04‐001‐1ACS; Biological Industries), penicillin and streptomycin (P1400; Solarbio). The cells were incubated at 37°C in a humidified atmosphere containing 5% CO_2_.

### 4.8 Cell transfection

The miRNA inhibitors, mimics, and negative controls were all obtained from RiboBio. First, an equal amount of cell suspension was pipetted into six‐well plates and incubated for 24 h. Then, the cells were transfected with miRNA inhibitors, mimics, or negative controls with Lipofectamine transfection reagent (Invitrogen). After 6 h, replace the transfection complex medium in the six‐well plate with fresh medium.

### 4.9 Colony formation assay

Briefly, 2 × 10^3^ cells/group transfected with miRNA inhibitors, mimics or negative controls were pipetted into six‐well plates and cultured in an incubator with 5% CO_2_ for 6−12 days with RPMI‐1640 medium supplemented with 10% FBS. The colonies were fixed using 4% formaldehyde (BL539A; Biosharp). Then, the colonies were stained with 0.05% crystal violet (548‐62‐9; Sigma Chemical Company) for 10 min. Finally, colonies were counted via a microscope (Olympus).

### 4.10 Wound healing assay

Briefly, 5 × 10^5^ cells were pipetted into six‐well plates and cultured using a medium containing 10% FBS. Subsequently, the cells were transfected with miRNA inhibitors, mimics, or negative controls, respectively. when the cells proliferated to 80% confluence, scrape the cell layer across each culture plate with a 200 μL sterile pipette tip to create a uniform straight wound. Subsequently, the cells were cultured with fresh medium without FBS. The inverted microscope (× 10) was used to record the extent of wound closures at 0 and 24 h. Finally, the width of the wound was tested by the Image‐Pro Plus 6.0 software.

### 4.11 Transwell migration and invasion assay

1 × 10^5^ KYSE‐30 cells, KYSE‐150 cells, or TE‐1 cells transfected with miRNA inhibitors, mimics, or negative controls were pipetted into the upper chambers (24‐well insert, 8 μm pore size; Corning Costar) with 200 μL RPMI‐1640 medium without FBS. Additionally, the lower chambers need to add 600 μL medium with 20% FBS as a chemo‐attractant. After 15−20 h incubation, cells on the upper side were wiped off with a cotton‐tipped swab, while the cells on the lower side were fixed with 4% formaldehyde and stained using 0.05% crystal violet for 10 min. The protocol of cell invasion assays was similar to the cell migration assay. The difference was the 200 μg/mL Matrigel (356234; Becton, Dickinson and Company) need to precoat at the transwell units overnight. The cells on the membrane of the lower side were considered as migrated/invaded cells and were photographed and counted at ×200 magnification.

### 4.12 Mass spectrometry analysis

Peptides (1 μg per sample) were loaded into a nanoflow HPLC Easy‐nLC1200 system (Thermo Fisher Scientific) for proteomic analysis. The Proteomic analyses were performed using a Q Exactive HF mass spectrometer (Thermo Fisher Scientific).

The raw data were researched in Proteome Discoverer 2.2 and the protein was identified using ion‐based label‐free quantitative methods. The significance was determined by the analysis of variance based on peptidome and protein levels. The Hemi (version 2.0) software was used to generate proteomic profiling.

### 4.13 Statistical analysis

All statistics were calculated using GraphPad Prism (version 8.3.0) and SPSS (IBM, version 22.0). Additionally, the abnormally expressed miRNAs in plasma/plasma‐derived EVs between the ESCC patients and HCs were compared using the Mann–Whitney *U* test.^12^ FC was calculated based on the median expression of the two groups of samples. FC > 1.5 or FC < 0.67, *p* value < 0.05 as the significant level. The ANOVA analysis was applied to compare the aberrantly expressed miRNAs in plasma/plasma‐derived EVs of ESCC patients with different LNM stages, *p* value < 0.05 was considered statistically significant. Heatmaps were generated using the heatmap package (version 3.5.1). An UpSet plot was also performed using the R package “UpSetR” (https://cran.r‐project.org/web/packages/UpSetR/). The Student's *t*‐test was performed for comparing the miRNA expression of ESCC and HC, and the miRNA expression was log‐transformed before *t*‐test was performed. Logistic regression analysis was applied to establish diagnostic panels consisting of candidate miRNAs. The ROC curves were plotted using each miRNA expression value. The AUC with a 95% confidence interval was calculated for each ROC curve. The GO and KEGG enrichment analysis of abnormally expressed proteins were performed by the DAVID website (https://david.ncifcrf.gov/). Pearson's correlation analysis was used to detect the correlation between plasma miRNA levels and plasma EVs‐derived miRNA levels in ESCC.

## AUTHOR CONTRIBUTIONS

L. B. Z., L. M. Z., L. L., W. Z., and Y. W. performed most of the experiments. Y. W., W. Z., X. W., H. B., X. L., and X. Y. analyzed the data. Y. W., W. Z., X. W., H. Z., and X. Y. conceived, designed, and supervised all studies. All authors have read and agreed to the published version of the manuscript.

## CONFLICT OF INTEREST STATEMENT

The authors declare that they have no any conflict of interest.

## ETHICS STATEMENT AND CONSENT TO PARTICIPATE

This study was reviewed and approved by the ethics committee of the Fourth Hospital of Hebei Medical University, and the number for the ethics approval is NO. 2022KS034.

## Supporting information

Supporting Information

## Data Availability

All data are available in the main text or the supplementary materials. All data generated in this study can be obtained from the corresponding authors upon reasonable request.
