# Supplementary material for: Identification of combinatorial miRNA panels derived from extracellular vesicles as biomarkers for esophageal squamous cell carcinoma
Source: MedComm (2020). 2023 Sep 18;4(5):e377. doi: 10.1002/mco2.377 (PMC10507283; doi:10.1002/mco2.377)
Supplement: Supplementary file 1 — Supporting Information [file MCO2-4-e377-s001.docx]

**Identification of combinatorial miRNA panels derived from extracellular vesicles as biomarkers for esophageal squamous cell carcinoma**

Yaojie Wang^1,2#^, Xiaoya Li^1,2#^, Xiaojian Wei^1,2^, Lei Li^1,2^, Hanyu Bai^1,2^, Xi Yan^2^, Hongtao Zhang^4^, Libo Zhao^1,2*^, Wei Zhou^3*^, and Lianmei Zhao^1,2 *^

**Affiliations:**

^1^ Research Center, the Fourth Hospital of Hebei Medical University, Jiankang Road 12, Shijiazhuang, 050011, China;

^2^ Key Laboratory of Tumor Gene Diagnosis, Prevention and Therapy; Clinical Oncology Research Center, Hebei Province, Shijiazhuang, 050011, China;

^3^ Hangzhou Institute of Medicine, Chinese Academy of Sciences, Hangzhou, 310022, China;

^4^University of Pennsylvania School of Medicine Philadelphia, 252 John Morgan Building 36th Street and Hamilton Walk, Philadelphia, PA 19104-6082, United States;

*** Correspondence:**

Lianmei Zhao, Research Center, the Fourth Hospital of Hebei Medical University; Key Laboratory of Tumor Gene Diagnosis, Prevention and Therapy of Hebei Province, Shijiazhuang, 050011, Hebei, China.

E-mails: zhaolianmei@hbydsy.com (L.M.Z.)

Wei Zhou, IHangzhou Institute of Medicine, Chinese Academy of Sciences, Hangzhou, 310022, China.

E-mails: zhouwei1989@iccas.ac.cn (W.Z.)

Libo Zhao, Research Center, the Fourth Hospital of Hebei Medical University; Key Laboratory of Tumor Gene Diagnosis, Prevention and Therapy of Hebei Province, Shijiazhuang, 050011, Hebei, China.

1. mails: lbzhao@iccas.ac.cn (L.B.Z.)

# Yaojie Wang and Xiaoya Li contributed equally to this work.

**Figure legends**

**Figure S1. Characterization of total plasma miRNAs and plasma EV-derived miRNAs.**

1. Distribution of mappable small RNAs in plasma and plasma-derived EVs.
2. The proportion of plasma EV-derived miRNAs in total plasma miRNAs.

**Figure S2. Screening of candidate miRNAs for ESCC diagnosis.**

A-B. Heatmap of aberrantly expressed miRNAs (HC *vs.* ESCC) in plasma (A) and plasma-derived EVs (B). FC > 1.5 or FC < 0.67, and *p* < 0.05

**Figure S3. EV-associated characteristic evaluation of candidate miRNAs.**

A. The proportion of miR-7641 in the top 10 EV fractions collected during SEC.

B. Relative expression levels of miR-636, miR-7641, miR-1246, and miR-28-3p detected from EVs enriched fraction samples with and without the pretreatment of Proteinase K and RNase A.

C. Pearson’s correlation analysis between plasma miRNA and plasma EVs-derived miRNA in ESCC (n = 22).

**Figure S4. The biological role of candidate miRNAs.**

A. The expression levels of miR-636, miR-7641, and miR-1246 were measured by qRT-PCR in KYSE-30, KYSE-150, and TE-1 cells transfected with the miRNA mimics or miRNA negative controls for 24 h.

B. The colony-formation ability of ESCC cells in control and transfection groups was detected by clonogenic assay. The number of colonies was calculated (n = 3) and plotted on a histogram.

C. The mobility ability of ESCC cells in control and transfection groups was detected by wound-healing assay.

D-E. The migration (D) and invasion (E) ability of ESCC cells in control and transfection groups were detected by Transwell and Matrigel assays, respectively. Average counts were collected from three random microscopic fields.

**p* < 0.05 and ***p* < 0.01 versus the control groups.

**Figure S1**

**
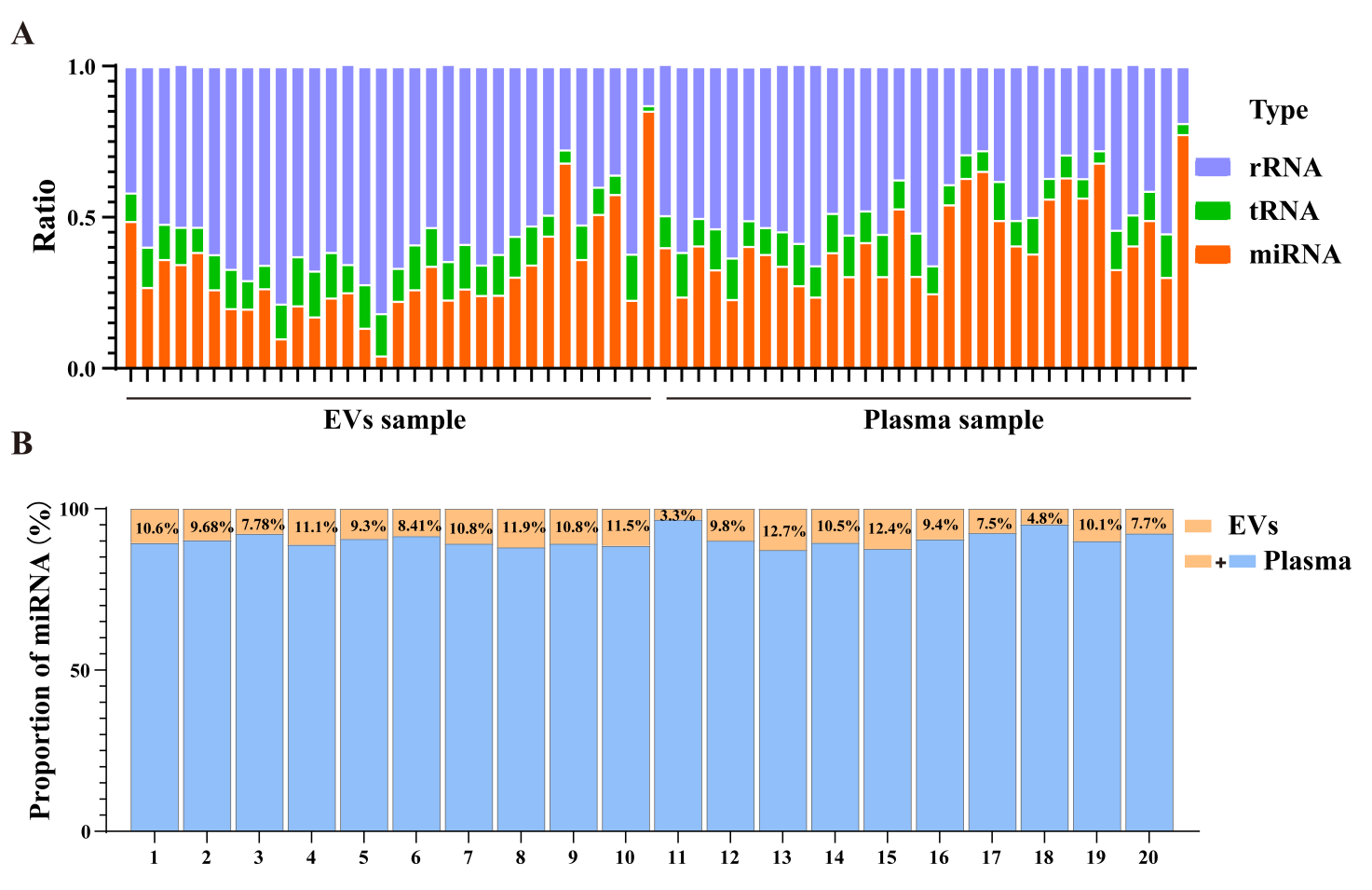
**

**Figure S2**

**
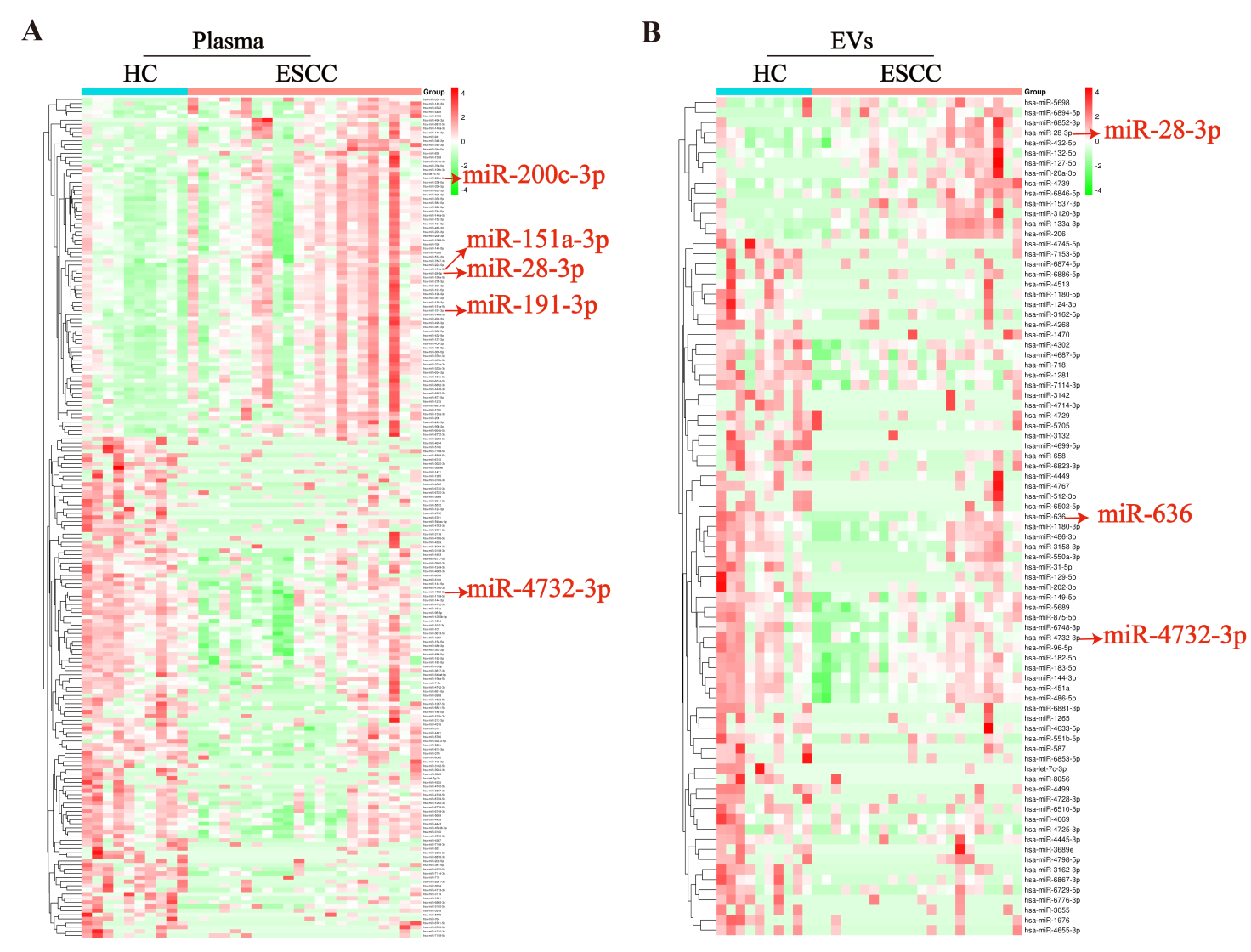
**

**Figure S3**

**
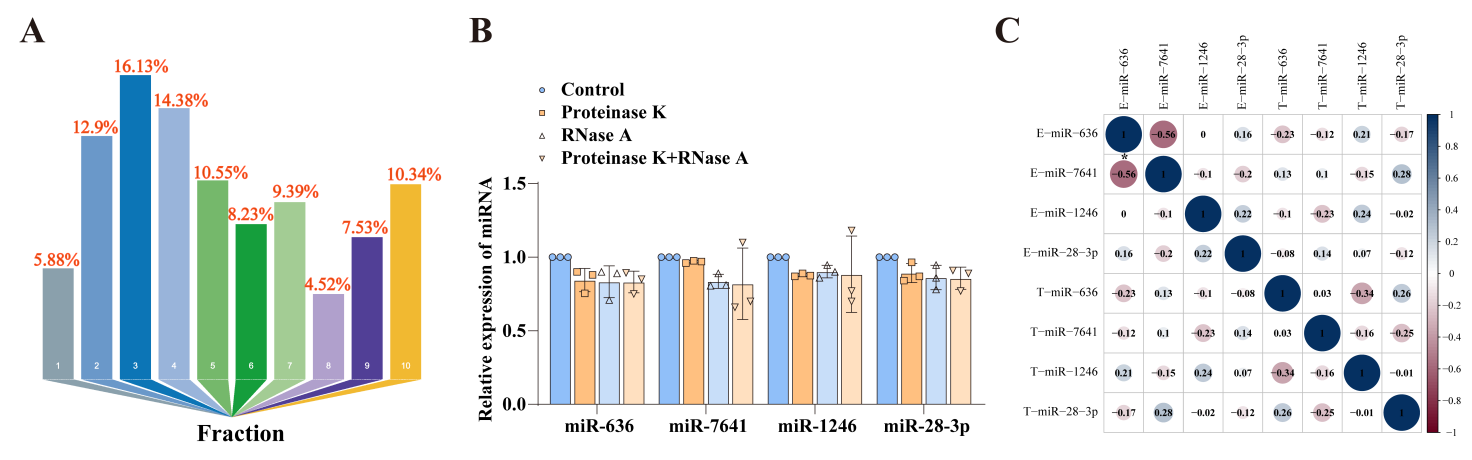
**

**Figure S4**

**
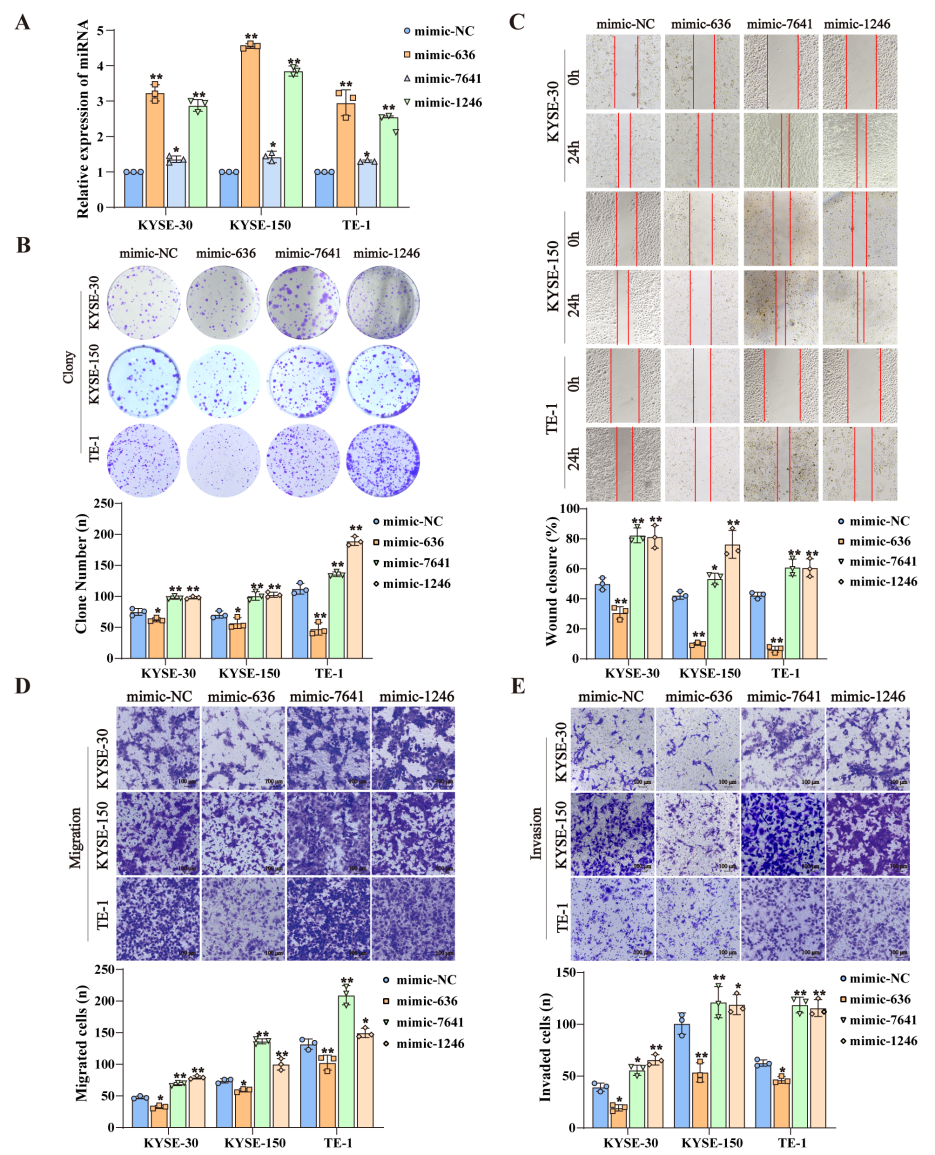
**

**Table S1. Clinical characteristics of patients with ESCC and healthy controls**

|  | **miRNA sequencing cohorts (N=32)** | **qRT-PCR discovery cohorts (N=40)** | **qRT-PCR training cohorts (N=100)** | **qRT-PCR test cohorts**  **(N=99)** |
| --- | --- | --- | --- | --- |
| **Group** |  |  |  |  |
| HC | 10 | 20 | 36 | 23 |
| ESCC | 22 | 20 | 64 | 76 |
| **Gender** |  |  |  |  |
| Male | 19 | 26 | 66 | 65 |
| Female | 13 | 14 | 34 | 34 |
| **Age (years)** | 60.34 ± 9.59 | 63.65 ± 9 | 57.65 ± 14.86 | 62.03 ± 14.12 |
| **T stage** |  |  |  |  |
| T1 | 7 | 9 | 33 | 37 |
| T2 | 4 | 8 | 5 | 8 |
| T3 | 11 | 2 | 20 | 24 |
| T4 | 0 | 1 | 6 | 7 |
| **N stage** |  |  |  |  |
| N0 | 13 | 9 | 32 | 34 |
| N1 | 5 | 5 | 18 | 24 |
| N2 | 3 | 4 | 7 | 12 |
| N3 | 1 | 2 | 7 | 6 |
| **Clinical stage** |  |  |  |  |
| I | 5 | 6 | 15 | 15 |
| II | 13 | 5 | 19 | 32 |
| III | 4 | 3 | 21 | 17 |
| Ⅳ | 0 | 6 | 9 | 12 |

**Table S2. Selection of ESCC diagnostic biomarkers from 204 miRNAs detected in plasma（HC *vs.* ESCC）**

| **ID** | **Source** | ***p* value** | **Log_2_FC** | **Regulation** |
| --- | --- | --- | --- | --- |
| hsa-miR-1275 | Plasma | 0.004994796 | Inf | up |
| hsa-miR-133b | Plasma | 0.00319479 | Inf | up |
| hsa-miR-299-5p | Plasma | 0.026624898 | Inf | up |
| hsa-miR-301b-3p | Plasma | 0.010212269 | Inf | up |
| hsa-miR-34b-3p | Plasma | 0.002034038 | Inf | up |
| hsa-miR-34c-3p | Plasma | 0.0122301 | Inf | up |
| hsa-miR-34c-5p | Plasma | 0.045968347 | Inf | up |
| hsa-miR-3591-5p | Plasma | 0.010661047 | Inf | up |
| hsa-miR-490-3p | Plasma | 0.008912846 | Inf | up |
| hsa-miR-495-3p | Plasma | 0.001194024 | Inf | up |
| hsa-miR-638 | Plasma | 0.020977168 | Inf | up |
| hsa-miR-6514-5p | Plasma | 0.030332996 | Inf | up |
| hsa-miR-6810-5p | Plasma | 0.040364617 | Inf | up |
| hsa-miR-6813-5p | Plasma | 0.014555156 | Inf | up |
| hsa-miR-6852-3p | Plasma | 0.002622556 | Inf | up |
| hsa-miR-206 | Plasma | 0.008143354 | 4.1506 | up |
| hsa-miR-487b-3p | Plasma | 0.030904872 | 3.8772 | up |
| hsa-miR-133a-3p | Plasma | 0.007740191 | 3.3076 | up |
| hsa-miR-4446-3p | Plasma | 3.52E-05 | 3.1350 | up |
| hsa-miR-432-5p | Plasma | 0.000915051 | 3.0530 | up |
| hsa-miR-409-3p | Plasma | 0.013703539 | 3.0320 | up |
| hsa-miR-4532 | Plasma | 0.008672424 | 2.9712 | up |
| hsa-miR-369-5p | Plasma | 0.04308663 | 2.9253 | up |
| hsa-miR-382-5p | Plasma | 0.002148554 | 2.9201 | up |
| hsa-miR-485-3p | Plasma | 0.019357967 | 2.8676 | up |
| hsa-miR-323b-3p | Plasma | 0.047478882 | 2.5978 | up |
| hsa-miR-493-3p | Plasma | 0.02316719 | 2.5210 | up |
| hsa-miR-485-5p | Plasma | 0.013602135 | 2.4228 | up |
| hsa-miR-134-5p | Plasma | 0.034657702 | 2.3484 | up |
| hsa-miR-99b-3p | Plasma | 0.008611161 | 2.3385 | up |
| hsa-miR-199a-5p | Plasma | 0.013703539 | 2.2913 | up |
| hsa-miR-328-3p | Plasma | 0.012076561 | 2.2761 | up |
| hsa-miR-625-3p | Plasma | 0.009309985 | 2.2701 | up |
| hsa-miR-381-3p | Plasma | 0.025333899 | 2.2644 | up |
| hsa-miR-6852-5p | Plasma | 0.008709286 | 2.2603 | up |
| hsa-miR-744-5p | Plasma | 0.02218534 | 2.2166 | up |
| hsa-miR-1468-5p | Plasma | 0.001983613 | 2.2013 | up |
| hsa-miR-199b-3p | Plasma | 0.02218534 | 2.1466 | up |
| hsa-miR-323a-3p | Plasma | 0.013732122 | 2.0686 | up |
| hsa-miR-877-5p | Plasma | 0.017184588 | 2.0566 | up |
| hsa-miR-584-5p | Plasma | 0.02218534 | 2.0294 | up |
| hsa-miR-181c-3p | Plasma | 0.024879099 | 2.0157 | up |
| hsa-miR-149-3p | Plasma | 0.032791723 | 2.0141 | up |
| **hsa-miR-191-3p** | **Plasma** | **0.004638934** | **1.9860** | **up** |
| hsa-miR-574-3p | Plasma | 0.013703539 | 1.9642 | up |
| hsa-miR-6772-3p | Plasma | 0.027849081 | 1.9475 | up |
| hsa-miR-330-3p | Plasma | 0.032249227 | 1.9051 | up |
| hsa-miR-224-5p | Plasma | 0.010617148 | 1.8928 | up |
| hsa-miR-4286 | Plasma | 0.032791723 | 1.8766 | up |
| hsa-miR-766-5p | Plasma | 0.00763388 | 1.8157 | up |
| hsa-miR-664b-5p | Plasma | 0.038113232 | 1.7969 | up |
| hsa-miR-127-3p | Plasma | 0.012076561 | 1.7386 | up |
| hsa-miR-941 | Plasma | 0.024882751 | 1.7316 | up |
| hsa-miR-1908-5p | Plasma | 0.027848204 | 1.7077 | up |
| hsa-miR-654-3p | Plasma | 0.047379443 | 1.6823 | up |
| hsa-miR-376c-3p | Plasma | 0.030641217 | 1.6628 | up |
| **hsa-miR-200c-3p** | **Plasma** | **0.001543614** | **1.6456** | **up** |
| hsa-miR-23b-5p | Plasma | 0.039878901 | 1.6030 | up |
| hsa-miR-139-3p | Plasma | 0.005361804 | 1.5988 | up |
| **hsa-miR-151a-3p** | **Plasma** | **0.001093312** | **1.5875** | **up** |
| hsa-miR-4488 | Plasma | 0.027989973 | 1.5635 | up |
| hsa-miR-760 | Plasma | 0.021603818 | 1.5080 | up |
| hsa-miR-27b-3p | Plasma | 0.004638934 | 1.3968 | up |
| hsa-miR-335-5p | Plasma | 0.015511382 | 1.3952 | up |
| **hsa-miR-28-3p** | **Plasma** | **0.002148554** | **1.3644** | **up** |
| hsa-miR-26b-3p | Plasma | 0.019391311 | 1.3483 | up |
| hsa-miR-145-5p | Plasma | 0.036211843 | 1.3310 | up |
| hsa-miR-361-3p | Plasma | 0.042776379 | 1.3308 | up |
| hsa-miR-6126 | Plasma | 0.03707979 | 1.3041 | up |
| hsa-miR-128-3p | Plasma | 0.047379443 | 1.2477 | up |
| hsa-miR-146a-5p | Plasma | 0.042776379 | 1.2370 | up |
| hsa-miR-152-3p | Plasma | 0.042776379 | 1.2196 | up |
| hsa-miR-151a-5p | Plasma | 0.015511382 | 1.2047 | up |
| hsa-let-7c-5p | Plasma | 0.009309985 | 1.1420 | up |
| hsa-miR-628-3p | Plasma | 0.01973548 | 1.0961 | up |
| hsa-miR-223-3p | Plasma | 0.034657702 | 1.0570 | up |
| hsa-miR-148a-3p | Plasma | 0.042776379 | 0.9745 | up |
| hsa-miR-30e-3p | Plasma | 0.015511382 | 0.9663 | up |
| hsa-miR-191-5p | Plasma | 0.038542236 | 0.8078 | up |
| hsa-miR-1538 | Plasma | 0.045250888 | 0.7967 | up |
| hsa-miR-7847-3p | Plasma | 0.034657702 | 0.7820 | up |
| hsa-miR-140-3p | Plasma | 0.047379443 | 0.7758 | up |
| hsa-miR-92a-2-5p | Plasma | 0.008405793 | -3.4557 | down |
| hsa-miR-651-5p | Plasma | 0.021613968 | -3.0487 | down |
| hsa-miR-3617-3p | Plasma | 0.033567125 | -2.8409 | down |
| hsa-miR-107 | Plasma | 0.009309985 | -2.8163 | down |
| hsa-miR-6729-5p | Plasma | 0.018288145 | -2.8120 | down |
| hsa-miR-4740-5p | Plasma | 0.037453617 | -2.7638 | down |
| hsa-miR-4520-5p | Plasma | 0.001728329 | -2.7510 | down |
| hsa-miR-96-5p | Plasma | 0.000118585 | -2.6809 | down |
| hsa-miR-5100 | Plasma | 0.00949164 | -2.5164 | down |
| hsa-miR-3158-3p | Plasma | 0.002667582 | -2.3850 | down |
| hsa-miR-4461 | Plasma | 0.019343232 | -2.3346 | down |
| hsa-miR-7114-3p | Plasma | 4.94E-05 | -2.2637 | down |
| hsa-miR-548au-3p | Plasma | 0.031483612 | -2.2341 | down |
| hsa-miR-5572 | Plasma | 0.023709126 | -2.1952 | down |
| hsa-miR-1249-3p | Plasma | 0.015190101 | -2.1275 | down |
| hsa-miR-615-3p | Plasma | 0.003607571 | -2.1014 | down |
| hsa-miR-451a | Plasma | 3.01E-06 | -1.9946 | down |
| hsa-miR-6821-5p | Plasma | 0.034244148 | -1.9702 | down |
| hsa-miR-1292-3p | Plasma | 0.002432329 | -1.9465 | down |
| hsa-miR-144-3p | Plasma | 0.001543614 | -1.8974 | down |
| hsa-miR-4738-5p | Plasma | 0.041854471 | -1.8907 | down |
| hsa-miR-144-5p | Plasma | 0.015552945 | -1.8638 | down |
| hsa-miR-320e | Plasma | 0.002136025 | -1.8287 | down |
| hsa-miR-4713-3p | Plasma | 0.012045214 | -1.7746 | down |
| hsa-miR-190a-5p | Plasma | 0.029474573 | -1.7425 | down |
| hsa-miR-4686 | Plasma | 0.012045214 | -1.7112 | down |
| hsa-miR-4742-3p | Plasma | 0.025933945 | -1.6511 | down |
| hsa-miR-3162-5p | Plasma | 0.030641217 | -1.5949 | down |
| hsa-miR-3613-5p | Plasma | 0.002950696 | -1.5509 | down |
| hsa-miR-4493 | Plasma | 0.007281399 | -1.5482 | down |
| hsa-miR-3940-3p | Plasma | 0.046168028 | -1.5102 | down |
| hsa-miR-6778-5p | Plasma | 0.024032913 | -1.5018 | down |
| hsa-miR-7-5p | Plasma | 0.004001411 | -1.4090 | down |
| hsa-miR-5689 | Plasma | 0.004119503 | -1.3962 | down |
| hsa-miR-1207-5p | Plasma | 0.007432425 | -1.3747 | down |
| **hsa-miR-4732-3p** | **Plasma** | **0.000762646** | **-1.3746** | **down** |
| hsa-miR-486-5p | Plasma | 0.000762646 | -1.3686 | down |
| hsa-miR-363-3p | Plasma | 0.013897565 | -1.3064 | down |
| hsa-miR-16-2-3p | Plasma | 0.004150443 | -1.3048 | down |
| hsa-miR-4259 | Plasma | 0.032647862 | -1.2736 | down |
| hsa-miR-4524b-5p | Plasma | 0.017287012 | -1.2667 | down |
| hsa-miR-375 | Plasma | 0.038542236 | -1.2417 | down |
| hsa-miR-3180 | Plasma | 0.034657702 | -1.1572 | down |
| hsa-miR-183-5p | Plasma | 0.00023332 | -1.1184 | down |
| hsa-miR-378i | Plasma | 0.026570284 | -1.1019 | down |
| hsa-miR-6748-3p | Plasma | 0.007689738 | -1.0915 | down |
| hsa-miR-3180-5p | Plasma | 0.031915496 | -1.0876 | down |
| hsa-miR-4443 | Plasma | 0.044151923 | -1.0852 | down |
| hsa-miR-4766-3p | Plasma | 0.039042821 | -1.0524 | down |
| hsa-miR-4732-5p | Plasma | 0.024882751 | -1.0444 | down |
| hsa-miR-182-5p | Plasma | 0.010617148 | -1.0385 | down |
| hsa-miR-15a-5p | Plasma | 0.02218534 | -1.0261 | down |
| hsa-miR-4316 | Plasma | 0.014555156 | -1.0158 | down |
| hsa-miR-6796-5p | Plasma | 0.013819087 | -1.0032 | down |
| hsa-miR-486-3p | Plasma | 0.003441641 | -0.9501 | down |
| hsa-miR-4685-3p | Plasma | 0.028377785 | -0.8948 | down |
| hsa-miR-4429 | Plasma | 0.040042993 | -0.8788 | down |
| hsa-miR-16-5p | Plasma | 0.047379443 | -0.8431 | down |
| hsa-miR-1255b-5p | Plasma | 0.029608866 | -0.6928 | down |
| hsa-miR-1199-5p | Plasma | 0.006180347 | -0.6645 | down |
| hsa-miR-1294 | Plasma | 0.029474573 | -0.6515 | down |
| hsa-miR-6777-5p | Plasma | 0.029608866 | -0.6373 | down |
| hsa-miR-1180-3p | Plasma | 0.040042993 | -0.5428 | down |
| hsa-let-7g-3p | Plasma | 0.029000358 | -Inf | down |
| hsa-miR-1205 | Plasma | 0.014334955 | -Inf | down |
| hsa-miR-124-3p | Plasma | 0.00054855 | -Inf | down |
| hsa-miR-1281 | Plasma | 0.003856405 | -Inf | down |
| hsa-miR-1471 | Plasma | 0.011515058 | -Inf | down |
| hsa-miR-188-5p | Plasma | 0.033540582 | -Inf | down |
| hsa-miR-192-3p | Plasma | 0.018782557 | -Inf | down |
| hsa-miR-193b-3p | Plasma | 0.023655154 | -Inf | down |
| hsa-miR-202-5p | Plasma | 0.012399083 | -Inf | down |
| hsa-miR-212-3p | Plasma | 0.023655154 | -Inf | down |
| hsa-miR-2681-3p | Plasma | 0.016426963 | -Inf | down |
| hsa-miR-3119 | Plasma | 0.002839667 | -Inf | down |
| hsa-miR-3176 | Plasma | 0.006696281 | -Inf | down |
| hsa-miR-3186-3p | Plasma | 0.048433736 | -Inf | down |
| hsa-miR-3200-5p | Plasma | 0.027707075 | -Inf | down |
| hsa-miR-3620-3p | Plasma | 0.000111965 | -Inf | down |
| hsa-miR-3668 | Plasma | 0.037386362 | -Inf | down |
| hsa-miR-3689e | Plasma | 0.016426963 | -Inf | down |
| hsa-miR-381-5p | Plasma | 0.033540582 | -Inf | down |
| hsa-miR-3934-3p | Plasma | 0.010564882 | -Inf | down |
| hsa-miR-3938 | Plasma | 0.033540582 | -Inf | down |
| hsa-miR-3944-3p | Plasma | 0.042224323 | -Inf | down |
| hsa-miR-3974 | Plasma | 0.046765304 | -Inf | down |
| hsa-miR-3978 | Plasma | 0.017528227 | -Inf | down |
| hsa-miR-422a | Plasma | 0.046422955 | -Inf | down |
| hsa-miR-4507 | Plasma | 0.035551769 | -Inf | down |
| hsa-miR-4535 | Plasma | 0.047839125 | -Inf | down |
| hsa-miR-4632-5p | Plasma | 0.024827991 | -Inf | down |
| hsa-miR-4634 | Plasma | 0.017110585 | -Inf | down |
| hsa-miR-4740-3p | Plasma | 0.008976089 | -Inf | down |
| hsa-miR-4763-3p | Plasma | 0.001755991 | -Inf | down |
| hsa-miR-4766-5p | Plasma | 0.047945955 | -Inf | down |
| hsa-miR-4792 | Plasma | 0.037485326 | -Inf | down |
| hsa-miR-5003-3p | Plasma | 0.040072626 | -Inf | down |
| hsa-miR-518b | Plasma | 0.000539959 | -Inf | down |
| hsa-miR-548at-5p | Plasma | 0.015879058 | -Inf | down |
| hsa-miR-557 | Plasma | 0.016150805 | -Inf | down |
| hsa-miR-5588-5p | Plasma | 0.000880578 | -Inf | down |
| hsa-miR-5701 | Plasma | 0.000456058 | -Inf | down |
| hsa-miR-5705 | Plasma | 0.010564882 | -Inf | down |
| hsa-miR-5706 | Plasma | 0.015528173 | -Inf | down |
| hsa-miR-6083 | Plasma | 0.029709536 | -Inf | down |
| hsa-miR-6086 | Plasma | 0.020953981 | -Inf | down |
| hsa-miR-6506-5p | Plasma | 0.012399083 | -Inf | down |
| hsa-miR-6720-3p | Plasma | 0.017498637 | -Inf | down |
| hsa-miR-6740-3p | Plasma | 0.016463489 | -Inf | down |
| hsa-miR-6745 | Plasma | 0.007604206 | -Inf | down |
| hsa-miR-6761-5p | Plasma | 0.046929523 | -Inf | down |
| hsa-miR-6763-3p | Plasma | 0.024760188 | -Inf | down |
| hsa-miR-6781-5p | Plasma | 0.005411548 | -Inf | down |
| hsa-miR-6865-3p | Plasma | 0.029090642 | -Inf | down |
| hsa-miR-6867-3p | Plasma | 0.029911315 | -Inf | down |
| hsa-miR-6876-3p | Plasma | 0.000456058 | -Inf | down |
| hsa-miR-7108-3p | Plasma | 0.013218571 | -Inf | down |
| hsa-miR-7108-5p | Plasma | 0.018782557 | -Inf | down |
| hsa-miR-7159-5p | Plasma | 0.048433736 | -Inf | down |
| hsa-miR-718 | Plasma | 0.001545128 | -Inf | down |
| hsa-miR-764 | Plasma | 0.022639802 | -Inf | down |
| hsa-miR-8058 | Plasma | 0.016403282 | -Inf | down |

**Table S3. Selection of ESCC diagnostic biomarkers from 83 miRNAs detected in plasma-derived EVs（HC *vs.* ESCC）**

| **ID** | **Source** | ***p* value** | **Log_2_FC** | **Regulation** |
| --- | --- | --- | --- | --- |
| hsa-miR-31-5p | EVs | 0.013595343 | -7.6952 | down |
| hsa-miR-3158-3p | EVs | 0.039441369 | -7.4732 | down |
| hsa-miR-718 | EVs | 0.005636571 | -7.1748 | down |
| hsa-miR-550a-3p | EVs | 0.040364617 | -6.7573 | down |
| hsa-miR-4513 | EVs | 0.035111364 | -6.7102 | down |
| hsa-miR-202-3p | EVs | 0.006696281 | -6.3966 | down |
| hsa-miR-551b-5p | EVs | 0.033633565 | -6.3688 | down |
| hsa-miR-6510-5p | EVs | 0.021669629 | -6.3477 | down |
| hsa-miR-658 | EVs | 0.005862512 | -6.1915 | down |
| hsa-miR-7153-5p | EVs | 0.007493424 | -6.0651 | down |
| hsa-miR-5705 | EVs | 0.031404993 | -6.0423 | down |
| hsa-miR-4445-3p | EVs | 0.030207752 | -5.9902 | down |
| hsa-miR-4655-3p | EVs | 0.021428465 | -5.9364 | down |
| hsa-miR-6867-3p | EVs | 0.008564837 | -5.9263 | down |
| hsa-miR-129-5p | EVs | 0.019409493 | -5.8803 | down |
| hsa-miR-4729 | EVs | 0.03551218 | -5.8737 | down |
| hsa-miR-1180-5p | EVs | 0.010130917 | -5.8633 | down |
| hsa-miR-4669 | EVs | 0.049782731 | -5.8250 | down |
| hsa-miR-1470 | EVs | 0.017528227 | -5.7573 | down |
| hsa-miR-3162-3p | EVs | 0.001545128 | -5.7212 | down |
| hsa-miR-1976 | EVs | 0.005210611 | -5.7212 | down |
| hsa-miR-1265 | EVs | 0.041398572 | -5.7212 | down |
| hsa-miR-3132 | EVs | 0.000225653 | -5.6713 | down |
| hsa-miR-6853-5p | EVs | 0.021428465 | -5.6713 | down |
| hsa-miR-6823-3p | EVs | 0.033540582 | -5.6713 | down |
| hsa-miR-4499 | EVs | 0.014334955 | -5.4830 | down |
| hsa-miR-4699-5p | EVs | 9.24E-05 | -5.4301 | down |
| hsa-miR-124-3p | EVs | 0.006098166 | -5.4148 | down |
| hsa-miR-6886-5p | EVs | 0.025649995 | -5.3966 | down |
| hsa-miR-4449 | EVs | 0.041938451 | -5.3688 | down |
| hsa-miR-4798-5p | EVs | 0.03140214 | -4.9610 | down |
| hsa-miR-6776-3p | EVs | 0.037091293 | -4.9199 | down |
| hsa-miR-4728-3p | EVs | 0.037887971 | -4.8259 | down |
| hsa-miR-8056 | EVs | 0.004167484 | -4.7086 | down |
| hsa-miR-4714-3p | EVs | 0.012399083 | -4.7086 | down |
| hsa-miR-4633-5p | EVs | 0.037091293 | -4.7086 | down |
| hsa-miR-6874-5p | EVs | 0.037091293 | -4.7086 | down |
| hsa-miR-587 | EVs | 0.042224323 | -4.7086 | down |
| hsa-miR-3689e | EVs | 0.012399083 | -4.6329 | down |
| hsa-miR-6502-5p | EVs | 0.02644497 | -4.6329 | down |
| hsa-miR-6881-3p | EVs | 0.014509924 | -4.0454 | down |
| hsa-miR-3142 | EVs | 0.019700707 | -4.0454 | down |
| hsa-miR-3655 | EVs | 0.021528852 | -4.0454 | down |
| hsa-miR-4745-5p | EVs | 0.021528852 | -4.0454 | down |
| hsa-miR-4268 | EVs | 0.032499451 | -4.0454 | down |
| hsa-let-7c-3p | EVs | 0.002332434 | -3.8759 | down |
| hsa-miR-512-3p | EVs | 0.016931473 | -3.8259 | down |
| hsa-miR-4767 | EVs | 0.032499451 | -3.8259 | down |
| hsa-miR-451a | EVs | 0.003441641 | -3.2349 | down |
| hsa-miR-3162-5p | EVs | 0.026397787 | -3.0869 | down |
| hsa-miR-6729-5p | EVs | 0.04561029 | -3.0793 | down |
| hsa-miR-96-5p | EVs | 0.005807476 | -2.4382 | down |
| **hsa-miR-636** | **EVs** | **0.002622556** | **-2.2779** | **down** |
| hsa-miR-486-3p | EVs | 0.032488235 | -2.0366 | down |
| **hsa-miR-4732-3p** | **EVs** | **0.008709286** | **-1.7902** | **down** |
| hsa-miR-144-3p | EVs | 0.024032913 | -1.7826 | down |
| hsa-miR-1180-3p | EVs | 0.035938649 | -1.5740 | down |
| hsa-miR-486-5p | EVs | 0.034657702 | -1.3186 | down |
| hsa-miR-4687-5p | EVs | 0.048549295 | -1.2502 | down |
| hsa-miR-149-5p | EVs | 0.016355147 | -1.2330 | down |
| hsa-miR-7114-3p | EVs | 0.008727747 | -1.2004 | down |
| hsa-miR-6748-3p | EVs | 0.039988277 | -1.1987 | down |
| hsa-miR-1281 | EVs | 0.001703093 | -1.1828 | down |
| hsa-miR-4725-3p | EVs | 0.040993118 | -1.1094 | down |
| hsa-miR-5689 | EVs | 0.013897565 | -1.0933 | down |
| hsa-miR-4302 | EVs | 0.026695394 | -1.0365 | down |
| hsa-miR-875-5p | EVs | 0.018783621 | -1.0159 | down |
| hsa-miR-183-5p | EVs | 0.015511382 | -0.8976 | down |
| hsa-miR-182-5p | EVs | 0.042776379 | -0.7210 | down |
| hsa-miR-3120-3p | EVs | 0.021841692 | 5.9932 | up |
| hsa-miR-127-5p | EVs | 0.037100532 | 5.7921 | up |
| hsa-miR-6894-5p | EVs | 0.035073636 | 5.7527 | up |
| hsa-miR-6846-5p | EVs | 0.04151797 | 5.7048 | up |
| hsa-miR-6852-3p | EVs | 0.016275159 | 5.5849 | up |
| hsa-miR-20a-3p | EVs | 0.031510496 | 4.6731 | up |
| hsa-miR-1537-3p | EVs | 0.039680182 | 4.1402 | up |
| hsa-miR-5698 | EVs | 0.04308663 | 2.9168 | up |
| hsa-miR-133a-3p | EVs | 0.021376182 | 2.8108 | up |
| hsa-miR-206 | EVs | 0.047379443 | 2.3857 | up |
| hsa-miR-432-5p | EVs | 0.031099184 | 1.7323 | up |
| **hsa-miR-28-3p** | **EVs** | **0.038542236** | **1.6572** | **up** |
| hsa-miR-132-5p | EVs | 0.035326025 | 0.8268 | up |
| hsa-miR-4739 | EVs | 0.042546552 | 0.7017 | up |

**Table S4. Selection of LNM diagnostic biomarkers from 118 miRNAs detected in ESCC plasma (HC *vs.* N0 *vs.* N1 *vs.* N2+3)**

|  |  | ***p* values for statistics** | | | |
| --- | --- | --- | --- | --- | --- |
| **miRNA ID** | **Source** | **Final *p* value** | **Bartlett *p* value** | **Kruskal *p* value** | **ANOVA *p* value** |
| hsa-miR-6830-5p | Plasma | 3.40E-05 | 0.299384826 | 0.001978202 | 3.40E-05 |
| hsa-miR-5191 | Plasma | 0.000395874 | 0 | 0.000395874 | 0.000750691 |
| hsa-miR-6739-5p | Plasma | 0.000954022 | 0.432793169 | 0.008400058 | 0.000954022 |
| **hsa-miR-1246** | **Plasma** | **0.001144811** | **0.207324598** | **0.00326442** | **0.001144811** |
| hsa-miR-6891-5p | Plasma | 0.002272861 | 0.354490529 | 0.004887495 | 0.002272861 |
| hsa-miR-6855-5p | Plasma | 0.002788054 | 0.794836017 | 0.023431191 | 0.002788054 |
| hsa-miR-6780b-5p | Plasma | 0.003065586 | 0.066051492 | 0.043840263 | 0.003065586 |
| hsa-miR-378i | Plasma | 0.003146787 | 0.434189191 | 0.017649441 | 0.003146787 |
| hsa-miR-3162-3p | Plasma | 0.0033612 | 0 | 0.0033612 | 0.000668557 |
| hsa-miR-3670 | Plasma | 0.003693764 | 0 | 0.003693764 | 0.000886171 |
| hsa-miR-4658 | Plasma | 0.003693764 | 0 | 0.003693764 | 0.001372392 |
| hsa-miR-210-5p | Plasma | 0.004739878 | 0 | 0.004739878 | 0.005809651 |
| hsa-miR-8086 | Plasma | 0.004960572 | 0.07110304 | 0.018519092 | 0.004960572 |
| hsa-miR-637 | Plasma | 0.0051734 | 0 | 0.0051734 | 0.000479642 |
| hsa-miR-200b-5p | Plasma | 0.005464146 | 5.68E-05 | 0.005464146 | 0.002734093 |
| hsa-miR-4644 | Plasma | 0.006799173 | 0.745390143 | 0.02182455 | 0.006799173 |
| hsa-miR-4461 | Plasma | 0.007447894 | 0.058498352 | 0.033039037 | 0.007447894 |
| hsa-miR-6831-5p | Plasma | 0.008027703 | 0.013226366 | 0.008027703 | 0.003477537 |
| hsa-miR-6499-5p | Plasma | 0.008392155 | 0.000527485 | 0.008392155 | 0.001335525 |
| hsa-miR-6861-3p | Plasma | 0.008392155 | 0.024431715 | 0.008392155 | 0.00030782 |
| hsa-miR-378f | Plasma | 0.00840207 | 0.35478244 | 0.02024663 | 0.00840207 |
| hsa-miR-6894-5p | Plasma | 0.008953306 | 0.000324745 | 0.008953306 | 0.089015945 |
| hsa-miR-152-5p | Plasma | 0.008966268 | 0 | 0.008966268 | 0.003465512 |
| hsa-miR-3678-3p | Plasma | 0.008966268 | 0 | 0.008966268 | 0.005078933 |
| hsa-miR-4740-3p | Plasma | 0.008966268 | 0 | 0.008966268 | 0.003465512 |
| hsa-miR-4676-5p | Plasma | 0.009003728 | 0.065529264 | 0.036257445 | 0.009003728 |
| hsa-miR-520d-5p | Plasma | 0.009796029 | 0.000244062 | 0.009796029 | 0.019442721 |
| hsa-miR-4516 | Plasma | 0.010266167 | 0.099060735 | 0.023577459 | 0.010266167 |
| hsa-miR-1268b | Plasma | 0.011165122 | 0.150335364 | 0.030218249 | 0.011165122 |
| hsa-miR-570-3p | Plasma | 0.011956357 | 0 | 0.011956357 | 0.006810439 |
| hsa-miR-487b-3p | Plasma | 0.013502929 | 0.004469139 | 0.013502929 | 0.814190879 |
| hsa-miR-580-3p | Plasma | 0.013642141 | 0 | 0.013642141 | 0.009605875 |
| hsa-miR-365b-3p | Plasma | 0.014317179 | 0.003048062 | 0.014317179 | 0.0073324 |
| hsa-miR-6884-5p | Plasma | 0.014595443 | 0.722183174 | 0.027855128 | 0.014595443 |
| hsa-miR-6514-3p | Plasma | 0.014603454 | 0.052297684 | 0.074371997 | 0.014603454 |
| hsa-miR-6833-5p | Plasma | 0.015693807 | 0.000455846 | 0.015693807 | 0.021317667 |
| hsa-miR-192-3p | Plasma | 0.016395127 | 0 | 0.016395127 | 0.009875455 |
| hsa-miR-544a | Plasma | 0.016395127 | 0 | 0.016395127 | 0.00723073 |
| hsa-miR-3127-5p | Plasma | 0.016507932 | 0.372107895 | 0.042737246 | 0.016507932 |
| hsa-miR-6751-5p | Plasma | 0.018450805 | 0.289984498 | 0.012245743 | 0.018450805 |
| hsa-miR-205-5p | Plasma | 0.018576862 | 0.075108617 | 0.02936262 | 0.018576862 |
| hsa-miR-5100 | Plasma | 0.018925005 | 0.144724851 | 0.029279115 | 0.018925005 |
| hsa-miR-1281 | Plasma | 0.020222088 | 0.052230677 | 0.038499333 | 0.020222088 |
| hsa-miR-539-3p | Plasma | 0.020611911 | 0.01134334 | 0.020611911 | 0.044960057 |
| hsa-miR-6760-3p | Plasma | 0.022234101 | 0.073830097 | 0.095755398 | 0.022234101 |
| hsa-miR-3680-3p | Plasma | 0.022782193 | 0 | 0.022782193 | 0.199120148 |
| hsa-miR-3187-3p | Plasma | 0.023518359 | 0.003088727 | 0.023518359 | 0.730516371 |
| hsa-miR-616-3p | Plasma | 0.02420537 | 0.007403601 | 0.02420537 | 0.660185876 |
| hsa-miR-5195-5p | Plasma | 0.024552756 | 0.263903765 | 0.047243323 | 0.024552756 |
| hsa-miR-5584-5p | Plasma | 0.025941143 | 0.000154002 | 0.025941143 | 0.838358052 |
| hsa-miR-501-3p | Plasma | 0.026355505 | 0.000307637 | 0.026355505 | 0.792616853 |
| hsa-miR-4660 | Plasma | 0.027975384 | 0.058536194 | 0.048484009 | 0.027975384 |
| hsa-miR-128-2-5p | Plasma | 0.028384707 | 0 | 0.028384707 | 0.078127028 |
| hsa-miR-137 | Plasma | 0.028384707 | 0 | 0.028384707 | 0.023627164 |
| hsa-miR-3164 | Plasma | 0.028384707 | 0 | 0.028384707 | 0.035462786 |
| hsa-miR-325 | Plasma | 0.028384707 | 0 | 0.028384707 | 0.022352213 |
| hsa-miR-372-3p | Plasma | 0.028384707 | 0 | 0.028384707 | 0.019410276 |
| hsa-miR-3926 | Plasma | 0.028384707 | 0 | 0.028384707 | 0.019410276 |
| hsa-miR-3934-3p | Plasma | 0.028384707 | 0 | 0.028384707 | 0.029781233 |
| hsa-miR-4322 | Plasma | 0.028384707 | 0 | 0.028384707 | 0.037523705 |
| hsa-miR-4465 | Plasma | 0.028384707 | 0 | 0.028384707 | 0.019410276 |
| hsa-miR-4690-5p | Plasma | 0.028384707 | 0 | 0.028384707 | 0.034727445 |
| hsa-miR-4768-3p | Plasma | 0.028384707 | 0 | 0.028384707 | 0.055417289 |
| hsa-miR-489-3p | Plasma | 0.028384707 | 0 | 0.028384707 | 0.022610775 |
| hsa-miR-513c-3p | Plasma | 0.028384707 | 0 | 0.028384707 | 0.019410276 |
| hsa-miR-6746-3p | Plasma | 0.028384707 | 0 | 0.028384707 | 0.037523705 |
| hsa-miR-6761-3p | Plasma | 0.028384707 | 0 | 0.028384707 | 0.022391909 |
| hsa-miR-6888-5p | Plasma | 0.028384707 | 0 | 0.028384707 | 0.019439256 |
| hsa-miR-7846-3p | Plasma | 0.028384707 | 0 | 0.028384707 | 0.019366256 |
| hsa-miR-8079 | Plasma | 0.028384707 | 0 | 0.028384707 | 0.019682848 |
| hsa-miR-876-5p | Plasma | 0.028384707 | 0 | 0.028384707 | 0.048862422 |
| hsa-miR-548q | Plasma | 0.029069675 | 0.072548061 | 0.049358698 | 0.029069675 |
| hsa-miR-33a-3p | Plasma | 0.030390607 | 0 | 0.030390607 | 0.023063667 |
| hsa-miR-4674 | Plasma | 0.030390607 | 0 | 0.030390607 | 0.148324006 |
| hsa-miR-4764-5p | Plasma | 0.030390607 | 0 | 0.030390607 | 0.058463206 |
| hsa-miR-499a-3p | Plasma | 0.030390607 | 0 | 0.030390607 | 0.025467534 |
| hsa-miR-8083 | Plasma | 0.030390607 | 0 | 0.030390607 | 0.039580962 |
| hsa-miR-382-3p | Plasma | 0.031801003 | 0.021244118 | 0.031801003 | 0.822427208 |
| hsa-miR-34c-5p | Plasma | 0.032347063 | 0.00979618 | 0.032347063 | 0.376055471 |
| hsa-miR-320b | Plasma | 0.032572793 | 0.033519906 | 0.032572793 | 0.739820607 |
| hsa-miR-449b-5p | Plasma | 0.032726435 | 0 | 0.032726435 | 0.013227606 |
| hsa-miR-181c-5p | Plasma | 0.033108275 | 0.003668877 | 0.033108275 | 0.89651488 |
| hsa-miR-4482-5p | Plasma | 0.034981701 | 0 | 0.034981701 | 0.034345023 |
| hsa-miR-3916 | Plasma | 0.03527553 | 0.016056529 | 0.03527553 | 0.037955278 |
| hsa-miR-10b-5p | Plasma | 0.036624854 | 0.076318677 | 0.081747521 | 0.036624854 |
| hsa-miR-4514 | Plasma | 0.037025821 | 5.50E-06 | 0.037025821 | 0.031990085 |
| hsa-miR-4669 | Plasma | 0.037997211 | 0 | 0.037997211 | 0.077197788 |
| hsa-miR-2116-3p | Plasma | 0.038061618 | 0.10902307 | 0.02791292 | 0.038061618 |
| hsa-miR-551a | Plasma | 0.038067591 | 0.009702809 | 0.038067591 | 0.050516096 |
| hsa-miR-429 | Plasma | 0.038183579 | 0.020783932 | 0.038183579 | 0.079269349 |
| hsa-miR-3615 | Plasma | 0.038456622 | 0.009863393 | 0.038456622 | 0.592677363 |
| hsa-miR-320a | Plasma | 0.038525084 | 0.000902277 | 0.038525084 | 0.804915307 |
| hsa-miR-4745-5p | Plasma | 0.039647525 | 0.014001451 | 0.039647525 | 0.44305755 |
| hsa-miR-3154 | Plasma | 0.040308481 | 0.960713572 | 0.056481421 | 0.040308481 |
| hsa-miR-106a-3p | Plasma | 0.040485949 | 0 | 0.040485949 | 0.5609777 |
| hsa-miR-3187-5p | Plasma | 0.040485949 | 0 | 0.040485949 | 0.243666927 |
| hsa-miR-4760-5p | Plasma | 0.040485949 | 0 | 0.040485949 | 0.020918731 |
| hsa-miR-5585-3p | Plasma | 0.040485949 | 0 | 0.040485949 | 0.461189911 |
| hsa-miR-6807-5p | Plasma | 0.040485949 | 0 | 0.040485949 | 0.332542711 |
| hsa-miR-744-3p | Plasma | 0.040485949 | 0 | 0.040485949 | 0.271883478 |
| hsa-miR-4762-3p | Plasma | 0.040735448 | 0.046899558 | 0.040735448 | 0.079176574 |
| hsa-miR-4731-3p | Plasma | 0.041380541 | 0 | 0.041380541 | 0.032970474 |
| hsa-miR-431-5p | Plasma | 0.041786204 | 0.014890445 | 0.041786204 | 0.96889411 |
| hsa-miR-193b-5p | Plasma | 0.041933659 | 0.03105909 | 0.041933659 | 0.128201611 |
| hsa-miR-369-3p | Plasma | 0.043196285 | 0.01321222 | 0.043196285 | 0.718256862 |
| hsa-miR-642b-5p | Plasma | 0.043785323 | 0.008115579 | 0.043785323 | 0.023075002 |
| hsa-miR-548az-5p | Plasma | 0.044361442 | 0 | 0.044361442 | 0.076232318 |
| hsa-miR-433-3p | Plasma | 0.04565413 | 0.028410976 | 0.04565413 | 0.962500428 |
| hsa-miR-4739 | Plasma | 0.045797076 | 0.135313553 | 0.057012516 | 0.045797076 |
| hsa-miR-4787-3p | Plasma | 0.046025106 | 0 | 0.046025106 | 0.599411061 |
| hsa-miR-1913 | Plasma | 0.046147262 | 0.454604126 | 0.099652805 | 0.046147262 |
| hsa-miR-3135a | Plasma | 0.047243323 | 0.000865211 | 0.047243323 | 0.072553682 |
| hsa-miR-1250-5p | Plasma | 0.047443651 | 0.000455536 | 0.047443651 | 0.506561608 |
| hsa-miR-6762-5p | Plasma | 0.047812162 | 0 | 0.047812162 | 0.017180805 |
| hsa-miR-185-5p | Plasma | 0.048586988 | 0.011308208 | 0.048586988 | 0.826724754 |
| hsa-miR-3619-3p | Plasma | 0.049293361 | 0 | 0.049293361 | 0.018785812 |
| hsa-miR-6850-3p | Plasma | 0.049293361 | 0 | 0.049293361 | 0.036787826 |
| hsa-miR-92b-3p | Plasma | 0.049644225 | 0.007619114 | 0.049644225 | 0.336639205 |

**Table S5. Selection of LNM diagnostic biomarkers from 108 miRNAs detected in ESCC plasma-derived EVs (HC *vs.* N0 *vs.* N1 *vs.* N2+3)**

|  |  | ***p* values for statistics** | | | |
| --- | --- | --- | --- | --- | --- |
| **miRNA ID** | **Source** | **Final *p* value** | **Bartlett *p* value** | **Kruskal *p* value** | **ANOVA *p* value** |
| hsa-miR-6133 | EVs | 0.000438618 | 0.849762556 | 0.011636506 | 0.000438618 |
| hsa-miR-6777-5p | EVs | 0.000967903 | 0.227324247 | 0.018470545 | 0.000967903 |
| hsa-miR-1255b-2-3p | EVs | 0.001629601 | 0 | 0.001629601 | 0.126366287 |
| hsa-miR-3919 | EVs | 0.001629601 | 0 | 0.001629601 | 4.76E-05 |
| hsa-miR-410-5p | EVs | 0.001679655 | 0.979898487 | 0.012166822 | 0.001679655 |
| hsa-miR-376b-5p | EVs | 0.003693764 | 0 | 0.003693764 | 0.000674985 |
| hsa-miR-455-3p | EVs | 0.003693764 | 0 | 0.003693764 | 0.000717182 |
| hsa-miR-4638-3p | EVs | 0.003693764 | 0 | 0.003693764 | 0.002748009 |
| hsa-miR-4640-3p | EVs | 0.003693764 | 0 | 0.003693764 | 0.000704293 |
| hsa-miR-3178 | EVs | 0.00478067 | 0.887266432 | 0.011535531 | 0.00478067 |
| hsa-miR-4516 | EVs | 0.006075627 | 0.17633495 | 0.018242444 | 0.006075627 |
| hsa-miR-6876-5p | EVs | 0.006603144 | 0 | 0.006603144 | 0.001648623 |
| hsa-miR-381-5p | EVs | 0.007248236 | 0 | 0.007248236 | 0.001677171 |
| hsa-miR-4739 | EVs | 0.008938928 | 0.111180315 | 0.026897276 | 0.008938928 |
| hsa-miR-492 | EVs | 0.008966268 | 0 | 0.008966268 | 0.013287758 |
| hsa-miR-519c-3p | EVs | 0.008966268 | 0 | 0.008966268 | 0.004092729 |
| hsa-miR-524-5p | EVs | 0.008966268 | 0 | 0.008966268 | 0.006215331 |
| hsa-miR-5571-3p | EVs | 0.008966268 | 0 | 0.008966268 | 0.005611897 |
| hsa-miR-4479 | EVs | 0.009298991 | 0.002498641 | 0.009298991 | 0.001058367 |
| hsa-miR-4298 | EVs | 0.009324832 | 0.446244553 | 0.0321149 | 0.009324832 |
| hsa-miR-6796-5p | EVs | 0.010276424 | 0.652456047 | 0.045554997 | 0.010276424 |
| hsa-miR-3125 | EVs | 0.010477072 | 0.019940911 | 0.010477072 | 0.50915246 |
| hsa-miR-6842-5p | EVs | 0.012722709 | 0 | 0.012722709 | 0.316383998 |
| hsa-miR-570-3p | EVs | 0.012798477 | 0 | 0.012798477 | 0.233589144 |
| hsa-miR-149-3p | EVs | 0.013592874 | 0.001495591 | 0.013592874 | 0.007104381 |
| hsa-miR-1915-3p | EVs | 0.013886188 | 0.065833723 | 0.023024576 | 0.013886188 |
| hsa-miR-7-1-3p | EVs | 0.015526417 | 0 | 0.015526417 | 0.270556742 |
| hsa-miR-365b-3p | EVs | 0.016298479 | 0.00060094 | 0.016298479 | 0.325718638 |
| hsa-miR-1243 | EVs | 0.016395127 | 0 | 0.016395127 | 0.150966533 |
| hsa-miR-4671-3p | EVs | 0.016395127 | 0 | 0.016395127 | 0.009013011 |
| hsa-miR-4749-5p | EVs | 0.016395127 | 0 | 0.016395127 | 0.005209848 |
| hsa-miR-500a-5p | EVs | 0.016395127 | 0 | 0.016395127 | 0.008595287 |
| hsa-miR-6859-3p | EVs | 0.016395127 | 0 | 0.016395127 | 0.01021461 |
| hsa-miR-7856-5p | EVs | 0.016395127 | 0 | 0.016395127 | 0.005549983 |
| hsa-miR-4439 | EVs | 0.016952057 | 0 | 0.016952057 | 0.032591305 |
| hsa-miR-1343-5p | EVs | 0.017251169 | 3.06E-08 | 0.017251169 | 0.138892745 |
| hsa-miR-1268b | EVs | 0.01823596 | 0.285453994 | 0.040660707 | 0.01823596 |
| hsa-miR-6126 | EVs | 0.018781308 | 0.010953305 | 0.018781308 | 0.030819109 |
| hsa-miR-3182 | EVs | 0.020030185 | 0.000238598 | 0.020030185 | 0.006347646 |
| hsa-miR-1268a | EVs | 0.020790939 | 0.558057524 | 0.017947912 | 0.020790939 |
| hsa-miR-133b | EVs | 0.021655329 | 0 | 0.021655329 | 0.168133936 |
| hsa-miR-6882-3p | EVs | 0.021655329 | 0 | 0.021655329 | 0.088047935 |
| hsa-miR-365a-3p | EVs | 0.021908969 | 0.038260693 | 0.021908969 | 0.025566634 |
| hsa-miR-3065-3p | EVs | 0.023573342 | 0.000402942 | 0.023573342 | 0.583108666 |
| hsa-miR-4804-5p | EVs | 0.023614445 | 0.034069532 | 0.023614445 | 0.0645911 |
| hsa-miR-4645-3p | EVs | 0.023925735 | 0.001998585 | 0.023925735 | 0.610884768 |
| hsa-miR-5090 | EVs | 0.024339581 | 0 | 0.024339581 | 0.141563098 |
| hsa-miR-2277-3p | EVs | 0.024501166 | 0.00648858 | 0.024501166 | 0.408823044 |
| hsa-miR-1237-5p | EVs | 0.02647956 | 0.613148475 | 0.044219365 | 0.02647956 |
| hsa-miR-556-3p | EVs | 0.026563335 | 0.071044464 | 0.011785307 | 0.026563335 |
| hsa-miR-4532 | EVs | 0.026904773 | 1.44E-05 | 0.026904773 | 0.203096511 |
| **hsa-miR-7641** | **EVs** | **0.028364212** | **0.284359048** | **0.067653309** | **0.028364212** |
| hsa-miR-1297 | EVs | 0.028384707 | 0 | 0.028384707 | 0.03429226 |
| hsa-miR-1321 | EVs | 0.028384707 | 0 | 0.028384707 | 0.019954331 |
| hsa-miR-1911-5p | EVs | 0.028384707 | 0 | 0.028384707 | 0.040421532 |
| hsa-miR-196b-3p | EVs | 0.028384707 | 0 | 0.028384707 | 0.044937468 |
| hsa-miR-215-3p | EVs | 0.028384707 | 0 | 0.028384707 | 0.03429226 |
| hsa-miR-3142 | EVs | 0.028384707 | 0 | 0.028384707 | 0.035377599 |
| hsa-miR-3677-3p | EVs | 0.028384707 | 0 | 0.028384707 | 0.020429996 |
| hsa-miR-4469 | EVs | 0.028384707 | 0 | 0.028384707 | 0.121714 |
| hsa-miR-4705 | EVs | 0.028384707 | 0 | 0.028384707 | 0.035377599 |
| hsa-miR-4782-3p | EVs | 0.028384707 | 0 | 0.028384707 | 0.019322015 |
| hsa-miR-644a | EVs | 0.028384707 | 0 | 0.028384707 | 0.020167436 |
| hsa-miR-648 | EVs | 0.028384707 | 0 | 0.028384707 | 0.02977452 |
| hsa-miR-6515-3p | EVs | 0.028384707 | 0 | 0.028384707 | 0.019360866 |
| hsa-miR-6718-5p | EVs | 0.028384707 | 0 | 0.028384707 | 0.044937468 |
| hsa-miR-6806-5p | EVs | 0.028384707 | 0 | 0.028384707 | 0.03429226 |
| hsa-miR-6880-3p | EVs | 0.028384707 | 0 | 0.028384707 | 0.019360866 |
| hsa-miR-6895-3p | EVs | 0.028384707 | 0 | 0.028384707 | 0.019782093 |
| hsa-miR-7843-3p | EVs | 0.028384707 | 0 | 0.028384707 | 0.019439981 |
| hsa-miR-7978 | EVs | 0.028384707 | 0 | 0.028384707 | 0.019782093 |
| hsa-miR-8062 | EVs | 0.028384707 | 0 | 0.028384707 | 0.019319788 |
| hsa-miR-6846-5p | EVs | 0.029050933 | 0.749727005 | 0.043050363 | 0.029050933 |
| hsa-miR-6127 | EVs | 0.029326555 | 0 | 0.029326555 | 0.005774747 |
| hsa-miR-1284 | EVs | 0.03038865 | 0 | 0.03038865 | 0.291945385 |
| hsa-miR-5047 | EVs | 0.030390607 | 0 | 0.030390607 | 0.066830875 |
| hsa-miR-199b-5p | EVs | 0.031064993 | 0.004266955 | 0.031064993 | 0.269351991 |
| hsa-miR-7107-5p | EVs | 0.032302691 | 0.29586547 | 0.122559025 | 0.032302691 |
| hsa-miR-3918 | EVs | 0.033857685 | 0 | 0.033857685 | 0.20198224 |
| hsa-miR-376a-3p | EVs | 0.034189474 | 0.000247329 | 0.034189474 | 0.715056703 |
| hsa-miR-4738-3p | EVs | 0.034426735 | 0.000422149 | 0.034426735 | 0.741717715 |
| hsa-miR-4658 | EVs | 0.034569937 | 0.05120106 | 0.098451457 | 0.034569937 |
| hsa-miR-4713-3p | EVs | 0.035467878 | 0.001231368 | 0.035467878 | 0.033270282 |
| hsa-miR-4493 | EVs | 0.035709037 | 0.064487651 | 0.077566546 | 0.035709037 |
| hsa-miR-4651 | EVs | 0.03585475 | 0.016108322 | 0.03585475 | 0.025350869 |
| hsa-miR-4725-3p | EVs | 0.036523513 | 0.269358905 | 0.065383742 | 0.036523513 |
| hsa-miR-378i | EVs | 0.03666918 | 0.015619479 | 0.03666918 | 0.069368625 |
| hsa-miR-6826-5p | EVs | 0.037374824 | 0 | 0.037374824 | 0.005388881 |
| hsa-miR-125b-5p | EVs | 0.04012647 | 0.000110137 | 0.04012647 | 0.334297802 |
| hsa-miR-3665 | EVs | 0.040172165 | 0 | 0.040172165 | 0.054831319 |
| hsa-miR-7106-5p | EVs | 0.040172165 | 0 | 0.040172165 | 0.041413469 |
| hsa-miR-140-3p | EVs | 0.040214408 | 0.000158652 | 0.040214408 | 0.77245214 |
| hsa-miR-3175 | EVs | 0.040485949 | 0 | 0.040485949 | 0.284489508 |
| hsa-miR-637 | EVs | 0.040485949 | 0 | 0.040485949 | 0.211979188 |
| hsa-miR-3662 | EVs | 0.041380541 | 0 | 0.041380541 | 0.036243184 |
| **hsa-miR-1246** | **EVs** | **0.042158154** | **0.018230006** | **0.042158154** | **0.002825518** |
| hsa-miR-1-3p | EVs | 0.043662935 | 0.000366565 | 0.043662935 | 0.485932968 |
| hsa-miR-4723-3p | EVs | 0.045701329 | 0.534821152 | 0.049330926 | 0.045701329 |
| hsa-miR-1247-5p | EVs | 0.045979794 | 0.001556569 | 0.045979794 | 0.426325648 |
| hsa-miR-8485 | EVs | 0.046052047 | 0.153584193 | 0.025451985 | 0.046052047 |
| hsa-miR-133a-3p | EVs | 0.046607893 | 0.000145161 | 0.046607893 | 0.361054004 |
| hsa-miR-4522 | EVs | 0.046661867 | 0.105366562 | 0.051514271 | 0.046661867 |
| hsa-miR-4286 | EVs | 0.046670952 | 0.000586572 | 0.046670952 | 0.623356433 |
| hsa-miR-4762-3p | EVs | 0.047812162 | 0 | 0.047812162 | 0.015140726 |
| hsa-miR-6781-5p | EVs | 0.04909749 | 0.24660983 | 0.107394746 | 0.04909749 |
| hsa-miR-4773 | EVs | 0.049293361 | 0 | 0.049293361 | 0.040008619 |
| hsa-miR-1343-3p | EVs | 0.049318895 | 0 | 0.049318895 | 0.509554218 |
| hsa-miR-99a-5p | EVs | 0.049434281 | 0.006359074 | 0.049434281 | 0.086807993 |

**Table S6. 25 miRNAs were co-existing in ESCC plasma and plasma-derived EVs**

| **ID** | **Source** | ***p* value** | **Log_2_FC** | **Source** | ***p* value** | **FDR** | **Log_2_FC** | **Regulation** |
| --- | --- | --- | --- | --- | --- | --- | --- | --- |
| hsa-miR-432-5p | Plasma | 0.0009 | 3.05 | EVs | 0.0311 | 0.79 | 1.73 | up |
| **hsa-miR-28-3p** | **Plasma** | **0.0021** | **1.36** | **EVs** | **0.0385** | **0.79** | **1.66** | **up** |
| hsa-miR-6852-3p | Plasma | 0.0026 | 7.03 | EVs | 0.0163 | 0.79 | 5.58 | up |
| hsa-miR-133a-3p | Plasma | 0.0077 | 3.31 | EVs | 0.0214 | 0.79 | 2.81 | up |
| hsa-miR-206 | Plasma | 0.0081 | 4.15 | EVs | 0.0474 | 0.81 | 2.39 | up |
| hsa-miR-6867-3p | Plasma | 0.0299 | -7.11 | EVs | 0.0086 | 0.79 | -5.93 | down |
| hsa-miR-1281 | Plasma | 0.0039 | -6.46 | EVs | 0.0017 | 0.79 | -1.18 | down |
| hsa-miR-718 | Plasma | 0.0015 | -6.06 | EVs | 0.0056 | 0.79 | -7.17 | down |
| hsa-miR-3689e | Plasma | 0.0164 | -6.01 | EVs | 0.0124 | 0.79 | -4.63 | down |
| hsa-miR-124-3p | Plasma | 0.0005 | -5.98 | EVs | 0.0061 | 0.79 | -5.41 | down |
| hsa-miR-5705 | Plasma | 0.0106 | -4.7 | EVs | 0.0314 | 0.79 | -6.04 | down |
| hsa-miR-6729-5p | Plasma | 0.0183 | -2.81 | EVs | 0.0456 | 0.81 | -3.08 | down |
| hsa-miR-96-5p | Plasma | 0.0001 | -2.68 | EVs | 0.0058 | 0.79 | -2.44 | down |
| hsa-miR-3158-3p | Plasma | 0.0027 | -2.39 | EVs | 0.0394 | 0.79 | -7.7 | down |
| hsa-miR-7114-3p | Plasma | 0 | -2.26 | EVs | 0.0087 | 0.79 | -1.2 | down |
| hsa-miR-451a | Plasma | 0 | -1.99 | EVs | 0.0034 | 0.79 | -3.23 | down |
| hsa-miR-144-3p | Plasma | 0.0015 | -1.9 | EVs | 0.024 | 0.79 | -1.78 | down |
| hsa-miR-3162-5p | Plasma | 0.0306 | -1.59 | EVs | 0.0264 | 0.79 | -3.09 | down |
| hsa-miR-5689 | Plasma | 0.0041 | -1.4 | EVs | 0.0139 | 0.79 | -1.09 | down |
| **hsa-miR-4732-3p** | **Plasma** | **0.0008** | **-1.37** | **EVs** | **0.0087** | **0.79** | **-1.79** | **down** |
| hsa-miR-486-5p | Plasma | 0.0008 | -1.37 | EVs | 0.0347 | 0.79 | -1.32 | down |
| hsa-miR-183-5p | Plasma | 0.0002 | -1.12 | EVs | 0.0155 | 0.79 | -0.9 | down |
| hsa-miR-6748-3p | Plasma | 0.0077 | -1.09 | EVs | 0.04 | 0.79 | -1.2 | down |
| hsa-miR-182-5p | Plasma | 0.0106 | -1.04 | EVs | 0.0428 | 0.79 | -0.72 | down |
| hsa-miR-486-3p | Plasma | 0.0034 | -0.95 | EVs | 0.0325 | 0.79 | -2.04 | down |

**Table S7. 10 miRNAs were co-existing in plasma and plasma-derived EVs of ESCC with different LNM stage**

| **ID** | **Source** | ***p* value** | **Source** | ***p* value** |
| --- | --- | --- | --- | --- |
| **hsa-miR-1246** | **Plasma** | **0.001144811** | **EVs** | **0.042158154** |
| hsa-miR-378i | Plasma | 0.003146787 | EVs | 0.03666918 |
| hsa-miR-4658 | Plasma | 0.003693764 | EVs | 0.034569937 |
| hsa-miR-637 | Plasma | 0.0051734 | EVs | 0.040485949 |
| hsa-miR-4516 | Plasma | 0.010266167 | EVs | 0.006075627 |
| hsa-miR-1268b | Plasma | 0.011165122 | EVs | 0.01823596 |
| hsa-miR-570-3p | Plasma | 0.011956357 | EVs | 0.012798477 |
| hsa-miR-365b-3p | Plasma | 0.014317179 | EVs | 0.016298479 |
| hsa-miR-4762-3p | Plasma | 0.040735448 | EVs | 0.047812162 |
| hsa-miR-4739 | Plasma | 0.045797076 | EVs | 0.008938928 |

**Table S8. Predicted consequential pairing of target region and miRNA**

| **Gene names** | **FC** | ***p* value** | **Predicted consequential pairing of target region (top) and miRNA (bottom）** | **Cumulative weighted context++ score** | **Total context++ score** |
| --- | --- | --- | --- | --- | --- |
| SRSF3 | 0.67 | 0.0026 | Position 2556-2563 f SRSF3 3' UTR 5' ...AAGUUGUAGUUUUACCAAGCACA...  miR-636 3' ...ACGCCCGCCCUGCUCGUUCGUGU... | 0 | -0.14 |
| CHD1 | 0.66 | 0.0049 | Position 1218-1224 of CHD1 3' UTR 5' ...GCAUUCUUACUCCCUCAAGCACU...  miR-636 3' ...ACGCCCGCCCUGCUCGUUCGUGU... | -0.01 | -0.09 |
| HK2 | 0.69 | 0.0078 | Position 1643-1650 of HK2 3' UTR 5' ...GUCCUAAAAUACAAACAAGCACA...  miR-636 3' ...ACGCCCGCCCUGCUCGUUCGUGU... | -0.25 | -0.25 |
| ANXA11 | 1.35 | 0.0020 | Position 1683-1689 of ANXA11 3' UTR 5' ...AGGUUGUAGUGAGCUGAGAUCAC...  miR-7641 3' ...CGAAUCGAAGGCUCUAGUU... | -0.01 | -0.05 |
| MYO1C | 1.25 | 0.0082 | Position 1002-1008 of MYO1C 3' UTR 5' ...GUUGGAAGACGCUGAGAGAUCAU...  miR-7641 3' ...CGAAUCGAAGGCUCUAGUU... | -0.04 | -0.04 |
| IPO7 | 1.24 | 0.0104 | Position 2291-2297 of IPO7 3' UTR 5' ...AUUAUAAUUUGGUAUAGAUCAAG...  miR-7641 3' ...CGAAUCGAAGGCUCUAGUU... | -0.01 | -0.02 |
| PKM | 1.22 | 0.0117 | Position 131-137 of PKM 3' UTR 5' ...GGACACCAGGGAAGAAGAUCAAC...  miR-7641 3' ...CGAAUCGAAGGCUCUAGUU... | -0.10 | -0.10 |
| CALU | 1.20 | 0.0203 | Position 474-481 of CALU 3' UTR 5' ...UAAUUUUGUAAGCCUGAGAUCAA...  miR-7641 3' ...CGAAUCGAAGGCUCUAGUU... | -0.28 | -0.30 |
| NDC1 | 1.25 | 0.0409 | Position 1166-1172 of NDC1 3' UTR 5' ...CCAUUCAUCUUCAUUAGAUCAAA...  miR-7641 3' ...CGAAUCGAAGGCUCUAGUU... | -0.01 | -0.03 |
| EPPK1 | 1.20 | 0.0003 | Position 992-998 of EPPK1 3' UTR 5' ...GCAGAACACAGCAAGAAUCCAUG...  miR-1246 3' ...GGACGAGGUUUUUAGGUAA... | 0 | -0.02 |
| ANXA4 | 1.27 | 0.0008 | Position 1257-1263 of ANXA4 3' UTR 5' ...GGGCGACAGAGCGAGAAUCCAUC...  miR-1246 3' ...GGACGAGGUUUUUAGGUAA... | 0 | -0.12 |
| ANXA11 | 1.31 | 0.0031 | Position 1200-1207 of ANXA11 3' UTR 5' ...GGUGUGAGGGAAGAGAAUCCAUA...  miR-1246 3' ...GGACGAGGUUUUUAGGUAA... | -0.01 | -0.10 |
| NT5E | 1.28 | 0.0358 | Position 310-316 of NT5E 3' UTR 5' ...UUCAUAUCCAUUUCUAAUCCAUC...  miR-1246 3' ...GGACGAGGUUUUUAGGUAA... | -0.12 | -0.19 |

**Table S9. Diagnostic performance of models containing 1 to 4 candidate miRNAs for ESCC**

| **Feature (plasma)** | **Train SN** | **Train SP** | **Train AUC** | **Test SN** | **Test SP** | **Test AUC** |
| --- | --- | --- | --- | --- | --- | --- |
| miR-636 | 92.2% | 36.1% | 0.689(0.58-0.798) | 89.5% | 26.1% | 0.571(0.436-0.706) |
| miR-7641 | 56.3% | 86.1% | 0.727(0.629-0.824) | 60.5% | 73.9% | 0.789(0.69-0.888) |
| miR-1246 | 40.6% | 91.7% | 0.611(0.501-0.72) | 40.8% | 91.3% | 0.633(0.523-0.743) |
| miR-28-3p | 84.4% | 30.6% | 0.592(0.478-0.706) | 17.1% | 95.7% | 0.507(0.395-0.62) |
| miR-636+miR-7641 | 100% | 33.3% | 0.783(0.694-0.872) | 97.4% | 26.1% | 0.744(0.639-0.849) |
| miR-636+miR-1246 | 82.8% | 47.2% | 0.712(0.609-0.816) | 75% | 43.5% | 0.655(0.536-0.774) |
| miR-636+miR-28-3p | 87.5% | 47.2% | 0.7(0.594-0.807) | 78.9% | 30.4% | 0.622(0.503-0.742) |
| miR-7641+miR-1246 | 59.4% | 88.9% | 0.711(0.612-0.811) | 64.5% | 78.3% | 0.771(0.678-0.864) |
| miR-7641+miR-28-3p | 51.6% | 97.2% | 0.716(0.618-0.814) | 60.5% | 95.7% | 0.764(0.671-0.857) |
| miR-1246+miR-28-3p | 46.9% | 88.9% | 0.618(0.509-0.726) | 53.9% | 87% | 0.658(0.556-0.76) |
| miR-636+miR-1246+miR-28-3p | 85.9% | 41.7% | 0.72(0.619-0.82) | 77.6% | 39.1% | 0.686(0.577-0.796) |
| miR-636+miR-7641+miR-28-3p | 100% | 33.3% | 0.792(0.705-0.878) | 97.4% | 26.1% | 0.764(0.665-0.863) |
| miR-636+miR-1246+miR-7641 | 62.5% | 86.1% | 0.796(0.71-0.881) | 59.2% | 82.6% | 0.755(0.654-0.855) |
| miR-7641+miR-1246+miR-28-3p | 60.9% | 86.1% | 0.709(0.609-0.81) | 65.8% | 73.9% | 0.769(0.678-0.861) |
| miR-636+miR-7641+miR-1246+miR-28-3p | 82.8% | 55.6% | 0.796(0.711-0.882) | 77.6% | 52.2% | 0.76(0.661-0.859) |
| **Feature (EVs)** | **Train SN** | **Train SP** | **Train AUC** | **Test SN** | **Test SP** | **Test AUC** |
| miR-636 | 92.2% | 52.8% | 0.683(0.553-0.813) | 80.3% | 34.8% | 0.555(0.393-0.718) |
| miR-7641 | 51.6% | 83.3% | 0.616(0.505-0.727) | 51.3% | 73.9% | 0.629(0.507-0.75) |
| miR-1246 | 60.9% | 72.2% | 0.641(0.531-0.752) | 65.8% | 60.9% | 0.658(0.522-0.793) |
| miR-28-3p | 45.3% | 91.7% | 0.628(0.518-0.813) | 46.1% | 95.7% | 0.72(0.615-0.824) |
| miR-636+miR-7641 | 95.3% | 47.2% | 0.753(0.655-0.852) | 84.2% | 39.1% | 0.68(0.55-0.81) |
| miR-636+miR-1246 | 92.2% | 55.6% | 0.737(0.622-0.853) | 84.2% | 43.5% | 0.602(0.453-0.751) |
| miR-636+miR-28-3p | 90.6% | 50% | 0.758(0.662-0.853) | 88.2% | 39.1% | 0.752(0.647-0.857) |
| miR-7641+miR-1246 | 79.7% | 69.4% | 0.776(0.68-0.872) | 71.1% | 56.5% | 0.747(0.636-0.857) |
| miR-7641+miR-28-3p | 45.3% | 88.9% | 0.602(0.492-0.711) | 50% | 82.6% | 0.693(0.59-0.797) |
| miR-1246+miR-28-3p | 60.9% | 86.1% | 0.743(0.648-0.838) | 51.3% | 95.7% | 0.748(0.653-0.843) |
| miR-636+miR-1246+miR-28-3p | 93.8% | 47.2% | 0.829(0.75-0.907) | 85.5% | 43.5% | 0.794(0.701-0.887) |
| miR-636+miR-7641+miR-28-3p | 95.3% | 41.7% | 0.756(0.661-0.851) | 90.8% | 39.1% | 0.756(0.653-0.86) |
| miR-636+miR-1246+miR-7641 | 93.8% | 55.6% | 0.82(0.732-0.908) | 80.3% | 43.5% | 0.76(0.649-0.872) |
| miR-7641+miR-1246+miR-28-3p | 65.6% | 83.3% | 0.767(0.675-0.859) | 57.9% | 82.6% | 0.759(0.664-0.854) |
| miR-636+miR-7641+miR-1246+miR-28-3p | 79.7% | 72.2% | 0.834(0.756-0.911) | 72.4% | 69.6% | 0.802(0.71-0.894) |

Abbreviations: SN, sensitivity; SP, specificity;

**Table S10. Diagnostic performance of models composed of candidate miRNAs for lymphatic node metastasis**

| **Feature（Plasma）** | **SN** | **SP** | **AUC** |
| --- | --- | --- | --- |
| miR-636 | 78.37% | 57.57% | 0.678(0.68-0.7) |
| miR-7641 | 67.56% | 57.57% | 0.602(0.61-0.63) |
| miR-636+miR-7641 | 83.78% | 50.00% | 0.685(0.67-0.73) |
| **Feature（EVs）** | **SN** | **SP** | **AUC** |
| miR-28-3p | 63.51% | 60.61% | 0.637(0.59-0.62) |
| miR-1246 | 89.19% | 56.06% | 0.743(0.74-0.82) |
| miR-28-3p+miR-1246 | 71.62% | 71.21% | 0.764(0.69-0.71) |
| **Feature（Plasma + EVs）** | **SN** | **SP** | **AUC** |
| T-miR-7641+E-miR-1246 | 83.78% | 51.52% | 0.738(0.68-0.73) |
| T-miR-636+E-miR-28-3p | 79.73% | 63.64% | 0.732(0.72-0.73) |
| T-miR-7641+E-miR-28-3p | 59.46% | 77.27% | 0.671(0.63-0.68) |
| T-miR-636+E-miR-1246 | 82.43% | 54.55% | 0.698(0.69-0.73) |
| T-miR-7641+E-miR-28-3p+E-miR-1246 | 83.78% | 68.18% | 0.785(0.76-0.78) |
| T-miR-636+E-miR-28-3p+E-miR-1246 | 63.51% | 83.33% | 0.768(0.68-0.72) |
| T-miR-7641+T-miR-636+E-miR-1246 | 78.38% | 60.61% | 0.74(0.7-0.71) |
| T-miR-7641+T-miR-636+E-miR-28-3p | 86.49% | 48.48% | 0.72(0.69-0.76) |
| T-miR-636+T-miR-7641+E-miR-28-3p+E-miR-1246 | 85.14% | 68.18% | 0.804(0.71-0.75) |

**Table S11. Sequences of primer and probe**

| **ID** | **Forward primers (5'-3'）** | **Reverse primers** | **Probes (5'-3'）** | **Stem-loop RT primers (5'-3'）** |
| --- | --- | --- | --- | --- |
| miR-4732-3p | CGCCCTGACCTGTCCT | GTGCAGGGTCCGAGGT | TCGCACTGGATACGACCAAGACAGGAC | GTCGTATCCAGTGCAGGGTCCGAGGTATTCGCACTGGATACGACCAAGAC |
| miR-96-5p | AGCTTTGGCACTAGCACATT | GTGCAGGGTCCGAGGT | GTATTCGCACTGGATACGACAAACGA | GTCGTATCCAGTGCAGGGTCCGAGGTATTCGCACTGGATACGACAAACGA |
| miR-636 | CTGTGCTTGCTCGTCCC | GTGCAGGGTCCGAGGT | TATTCGCACTGGATACGACTGCGGG | GTCGTATCCAGTGCAGGGTCCGAGGTATTCGCACTGGATACGACTGCGGG |
| miR-7641 | CGCGTTGATCTCGGAAG | GTGCAGGGTCCGAGGT | TAAGCGTCGTATCCAGTGCGAA | GTCGTATCCAGTGCAGGGTCCGAGGTATTCGCACTGGATACGACGCTTAG |
| miR-1246 | ACGGAGCGAATGGATTTTTGG | GTGCAGGGTCCGAGGT | CAGAGCCACCTGGGCAATTT | CAGTGCAGGGTCCGAGGTCAGAGCCACCTGGGCAATTTTTTTTTTTCCTGC |
| miR-28-3p | ACGGAAGGAGCTCACAGT | GTGCAGGGTCCGAGGT | TTCGCACTGGATACGACCTCAATAG | GTCGTATCCAGTGCAGGGTCCGAGGTATTCGCACTGGATACGACCTCAAT |
| miR-191-3p | CGGGCGCTGCGCTTGGATTT | CAGCCACAAAAGAGCACAAT | TTCAGGAGACAACAGG | CCTGTTGTCTCCAGCCACAAAAGAGCACAATATTTCAGGAGACAACAGGGGGGACG |
| miR-151a-3p | ACCGCTAGACTGAAGCTCC | GTGCAGGGTCCGAGGT | TTCGCACTGGATACGACCCTCAA | GTCGTATCCAGTGCAGGGTCCGAGGTATTCGCACTGGATACGACCCTCAA |
| miR-200c-3p | ACGCTAATACTGCCGGGTAAT | GTGCAGGGTCCGAGGT | TTCGCACTGGATACGACTCCATC | GTCGTATCCAGTGCAGGGTCCGAGGTATTCGCACTGGATACGACTCCATC |
| U6 | CTCGCTTCGGCAGCACA | GTGCAGGGTCCGAGGT | AGAAGATTAGCATGGCCCCTGCGCA | GTGCAGGGTCCGAGGT |
